# Evaluation of linear models and missing value imputation for the analysis of peptide-centric proteomics

**DOI:** 10.1186/s12859-019-2619-6

**Published:** 2019-03-14

**Authors:** Philip Berg, Evan W. McConnell, Leslie M. Hicks, Sorina C. Popescu, George V. Popescu

**Affiliations:** 1 Department of Biochemistry, Molecular Biology, Entomology, and Plant Pathology, Mississippi State University, Mississippi State, MS USA; 2 Institute for Genomics, Biocomputing and Biotechnology, Mississippi State University, Mississippi State, MS USA; 3 Department of Chemistry, University of North Carolina at Chapel Hill, Chapel Hill, NC USA; 4 The National Institute for Laser, Plasma & Radiation Physics, Bucharest, Romania

**Keywords:** Post-translational modifications, Redox proteome, Mass spectrometry, Multiple imputation, Linear regression models

## Abstract

**Background:**

Several methods to handle data generated from bottom-up proteomics via liquid chromatography-mass spectrometry, particularly for peptide-centric quantification dealing with post-translational modification (PTM) analysis like reversible cysteine oxidation are evaluated. The paper proposes a pipeline based on the R programming language to analyze PTMs from peptide-centric label-free quantitative proteomics data.

**Results:**

Our methodology includes variance stabilization, normalization, and missing data imputation to account for the large dynamic range of PTM measurements. It also corrects biases from an enrichment protocol and reduces the random and systematic errors associated with label-free quantification. The performance of the methodology is tested by performing proteome-wide differential PTM quantitation using linear models analysis (*limma*). We objectively compare two imputation methods along with significance testing when using multiple-imputation for missing data.

**Conclusion:**

Identifying PTMs in large-scale datasets is a problem with distinct characteristics that require new methods for handling missing data imputation and differential proteome analysis. Linear models in combination with multiple-imputation could significantly outperform a *t*-test-based decision method.

**Electronic supplementary material:**

The online version of this article (10.1186/s12859-019-2619-6) contains supplementary material, which is available to authorized users.

## Background

Covalent post-translational modifications (PTMs) have a significant impact on protein function and activity, while greatly increasing proteome complexity of an organism. Enzyme-catalyzed PTMs, such as acetylation, phosphorylation, or ubiquitination, can occur at one or multiple amino acid residues in a nascent protein following translation and folding, or on mature proteins as part of signal transduction pathways or regulatory/control processes [1]. PTMs modify the activation state of enzymes, change the subcellular localization, or modify the stability of proteins. The frequency with which PTMs occur, stoichiometry, timing, and location within a protein and the cell are crucial aspects for understanding the function of proteins and linking the dynamics of the whole proteome with physiological and pathological phenotypes of an organism [2].

Advances in mass spectrometry (MS) have accelerated the identification of PTM sites with high resolution in proteomes, where thousands of modifications are now routinely discovered following enrichment and quantitative methods [3]. Technological progress has also prompted the development of new protocols for the quantitative analysis of PTMs. Most published work has focused on detecting and quantifying well-known, classical PTMs such as phosphorylation, glycosylation, ubiquitination, and acetylation [4–9]. However, research on new types of PTMs is emerging; specifically, the study of redox-mediated PTMs is currently the focus of numerous recent studies as a basis to understand the roles that thiol oxidation play in cellular signaling [10–12]. Reactive oxygen species (ROS) produced intracellularly under physiological conditions as metabolic byproducts or in response to various environmental stress factors can directly affect proteins. The sulfur atom in cysteine and methionine residues of proteins are primarily susceptible to oxidation by ROS [13]. Oxidized intermediates of cysteine and methionine have important catalytic roles in the active site of some enzymes, or can affect the functions of ROS-sensitive proteins [14].

Developing approaches to analyze mass spectrometry data both accurately and comprehensively is a challenge in proteomics and a considerable bottleneck in determining biological significance. In particular, quantitative analysis of protein PTMs via bottom-up proteomics in various organisms or experimental systems remains a challenge. The main issues in PTM quantification and analysis are twofold. Biological factors including a low abundance of modified proteins and the transitory nature of PTMs [15] decrease experimental reproducibility and hamper their comprehensive spatial and temporal analysis. Also, technical factors, such as the intrinsic variability of PTM enrichment protocols and sensitivity in PTM detection where modified peptides may be more difficult to identify from their fragmentation spectra than unmodified counterparts [16], further influence the reproducibility of PTM identification and quantification. These challenges result in measurements with a large proportion of missing data, technical errors, batch biases, and data sets difficult to normalize and variance-stabilize. Additional constraints are identified in redox proteomics, including technical problems like sample handling and preparatory issues that can artificially shift the oxidation status of proteins [17] and difficulties in the post-MS quantitative analysis, where precise stoichiometric information is required to characterize dynamic and transient redox proteomes [18].

**Fig. 1:**
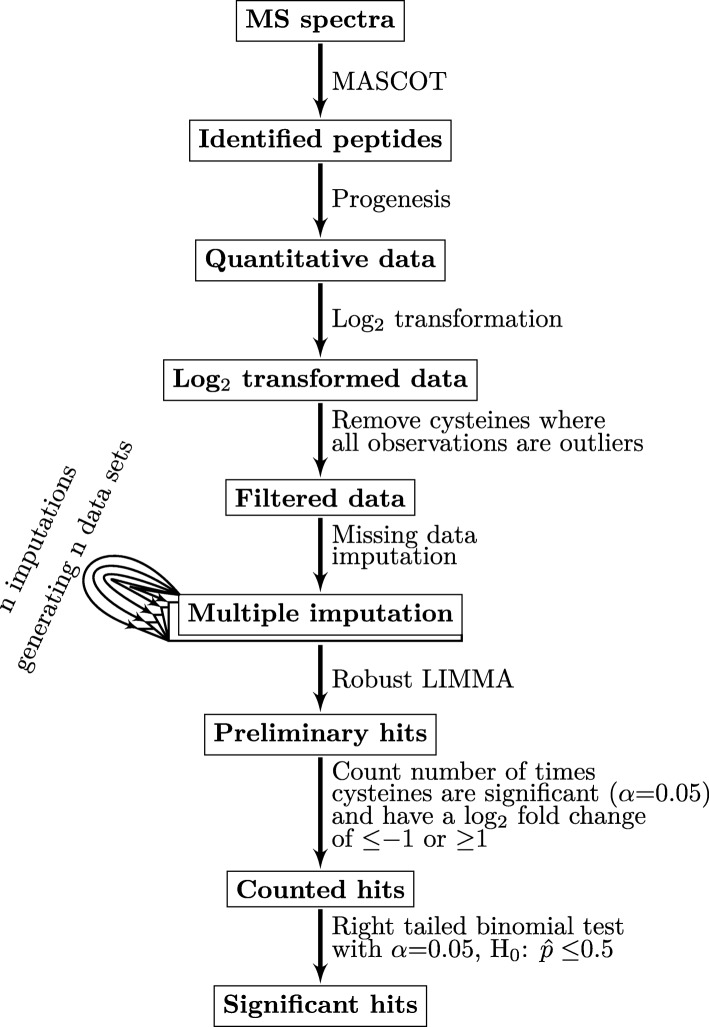
Schematic representation of the computational pipeline

To overcome known challenges in PTM quantification in large-scale datasets, we sought to develop data processing tools that would enable a more rigorous analysis of PTMs. Improved analysis of PTM datasets can significantly impact the biological insights of a study as well as provide new ways to improve proteomics. A performance evaluation of existing label-free MS quantification methods and software packages using a proteomic standard composed of an equimolar mixture of 48 human proteins (UPS1) spiked at different concentrations into a background of yeast cell lysate was presented in [19]. In this study, we describe a statistical methodology to address challenges related to peptide-centric identification and analysis. We developed a data analysis pipeline (Fig. 1) built on output from Progenesis QI for proteomics that is extendable to any label-free quantitation software using area-under-the-curve measurements. The pipeline consists of 1) a quantitation script, 2) an imputation method using sampling from a normal distribution with parameters robustly estimated from analyzed data, 3) a robust regression decision method based on *limma*, and 4) multiple imputation analysis. We provide a comparative analysis of linear modeling versus a Student’s *t*-test to detect relative abundance changes in two benchmark datasets: (1) Universal Proteomics Standard Set 1 (UPS1) and (2) enrichment of protein cysteines from the yeast proteome, both spiked into a complex protein lysate from the model green alga *Chlamydomonas reinhardtii* (Chlamydomonas) at different concentrations. We compare our imputation method using an empirical distribution sampling for missing data, multiple imputation and binomial testing against Random Forest (RF), a bootstrapping-based machine learning approach previously reported as a top performer in MS data imputation [20]. We found that robust estimation of the missing data distribution parameters combined with linear modeling and binomial testing performs better than all other combination of methods for the analysis of peptide-centric datasets.

## Materials

### Protein extraction for background lysate

Steps taken to culture wild-type *Chlamydomonas reinhardtii* CC-2895 and extract proteins were identical to our previous study [21]. For samples used to enrich reversible oxidation, iodoacetamide (IAM) was added to the lysis buffer to alkylate reduced cysteines. Final lysates in the global proteomics and reversible oxidation studies were suspended in 50 mM Tris, pH 8.0 with 0.5% SDS and 8 M urea at 1 mg/mL concentration.

### Spiking standard proteins/proteomes into background lysate

The Universal Proteomics Standard Set 1 (UPS1) was purchased from Sigma-Aldrich (St. Louis, MO, USA). For the global proteomics study, a vial of UPS1 containing 5 pmol each of 48 human proteins was resuspended in 50 μL of lysate to make an initial stock concentration of 100 fmol UPS1 per 1 μg lysate. Serial dilutions were performed twice to prepare both 50 and 25 fmol/μg UPS1. Each sample had a final volume of 25 μL and corresponded to roughly 25 μg total protein in subsequent processing. There were four technical replicates for each UPS1 concentration.

Intact Mass Spec-Compatible Yeast Protein Extract was from Promega (Madison, WI, USA). For the reversible oxidation study, a vial containing 1 mg of yeast proteome was resuspended in 1 mL of lysate to make an initial stock concentration of 1000 ng yeast per 1 μg lysate. A ten-fold dilution was performed by adding 100 μL stock to 900 μL lysate to make 100 ng/μg yeast. This sample was then two-fold diluted in 500 μL of lysate for a 50 ng/μg yeast. Each sample had a final volume of 500 μL and corresponded to roughly 500 μg total protein in subsequent processing. There were three technical replicates for each yeast proteome concentration.

### Preparation of samples for bottom-up proteomics

Steps taken for protein-level reversibly oxidized cysteine enrichment and subsequent LC-MS/MS analysis have been described previously [21]. For global proteomics, each sample was held at 30 °C and reduced using 10 mM dithiothreitol (DTT) for 1 h, alkylated with 30 mM IAM for 1 h, and then diluted four-fold with 75 μL of 50 mM Tris, pH 8. Proteins were digested with 1 μg of Trypsin Gold (Promega) for 16 h at 25 °C before quenching with 5 μL of 5% TFA. Following solid-phase extraction and vacuum centrifugation, samples were resuspended in 125 μL of water with 0.1% TFA before LC-MS/MS analysis identical to the reversible oxidation study.

## Methods

### Database searching and label-free quantification

Acquired spectral files (*.wiff) were imported into Progenesis QI for proteomics (Nonlinear Dynamics, version 2.0). A reference spectrum was automatically assigned, and total ion chromatograms were then aligned to minimize run-to-run differences in peak retention time. Each sample received a unique factor to normalize all peak abundance values resulting from systematic experimental variation. Alignment was validated (> 80% score) and a combined peak list (*.mgf) for all runs was exported for peptide sequence determination and protein inference by Mascot (Matrix Science, version 2.5.1). Database searching was performed against a combined database (19,603 entries total) containing *C. reinhardtii* JGI v5.5 proteins (https://phytozome.jgi.doe.gov/pz/portal.html; downloaded June 2016) and entries from the NCBI chloroplast (BK000554.2) and mitochondrial (NC_001638.1) databases. Sequences for either the 48 UPS1 proteins (www.sigmaaldrich.com/content/dam/sigma-aldrich/life-science/proteomics-and-protein/ups1-ups2-sequences.fasta; downloaded May 2016) or 6721 yeast proteins from UniProtKB (UP000002311; downloaded April 2016) were appended to the database. Searches of MS/MS data used a trypsin protease specificity with the possibility of two missed cleavages, peptide/fragment mass tolerances of 15 ppm/0.1 Da, and variable modifications of acetylation at the protein N-terminus and oxidation at methionine. Carbamidomethylation at cysteine was a fixed modification for global proteomics and variable for the reversible oxidation study. Significant peptide identifications above the identity or homology threshold were adjusted to less than 1% peptide FDR using the embedded Percolator algorithm [22] and uploaded to Progenesis for peak matching. Identifications with a score less than 13 were removed from consideration in Progenesis before exporting ‘peptide measurements’ and ‘protein measurements’ from the ‘Review Proteins’ stage. The mass spectrometry proteomics data have been deposited to the ProteomeXchange Consortium (http://proteomecentral.proteomexchange.org/cgi/GetDataset) via the PRIDE partner repository [23].

### Data pre-processing

Upon analyzing raw spectral files in Progenesis, we then prepared data from the global proteomics experiment (UPS1) for statistical analysis following the steps described below (implemented in Additional file 1). Our dataset initially consisted of 15,234 peptides matched to MS1 features in the peptide measurements export. After filtering for Mascot score above 13 and removing hits to the contaminant database, 13,452 peptides remained. Some features were matched with peptides having identical sequence, modifications, and Mascot score, but alternate protein accessions. These groups were reduced to satisfy the principle of parsimony [24] and represented by the protein accession with the highest number of unique peptides, else the protein with the largest confidence score assigned by Progenesis. Additionally, certain features were duplicated with differing peptide identifications and were reduced to a single peptide with the highest Mascot score. These steps reduced the dataset to 12,792 peptides. Identifiers were formed by concatenating protein accession to the peptide sequence, and duplicate identifiers were binned together. The final dataset consisted of 10,599 identifiers (10,207 from Chlamydomonas and 392 from UPS1 proteins).

Our dataset from the reversible oxidation study consisted of 4388 peptides initially and underwent similar processing steps (implemented in Additional file 2). Filtering for Mascot score and removing contaminants left 3752 peptides. We then consolidated groups with duplicate peak features, reducing the dataset to 3547 peptides. For this study, results were limited to only peptides with one or more Cys-sites of reversible oxidation, defined here as the absence of carbamidomethylation on at least one cysteine residue in the peptide sequence. This filter left 3162 peptides with previous sites of reversible cysteine oxidation. An identifier was then made by joining the protein accession of each feature with the particular site of Cys-oxidation in the protein sequence. Data was then reduced to unique identifiers by summing the abundance of all contributing features (i.e., peptide charge states, missed cleavages, and combinations of additional variable modifications). Each group was represented by the peptide with the highest Mascot score, leaving 2235 identifiers for statistical evaluation (1786 from Chlamydomonas and 449 from yeast).

### Data analysis pipeline

The first step in our pipeline is *performing a variance-stabilizing* transformation by taking the log_2_ of each measurement. *We then* remove features where all samples are outliers (defined here as observations outside the Tukey’s fences [25] with a *k* value of 1.5). Next, we implement a missing data imputation method using random sampling from a normal distribution with parameters robustly estimated from the entire dataset. We perform imputation by splitting up the features into two sets *X*^*w*^ and *X*^*wo*^, either with or without missing values, respectively. In matrix terms, the observations are described by:

1 $$ {X}^s=\left[\begin{array}{ccc}{x}_{11}^s& \cdots & {x}_{1M}^s\\ {}\vdots & \ddots & \vdots \\ {}{x}_{N1}^s& \cdots & {x}_{N\mathrm{M}}^s\end{array}\right] $$

where ${x}_{ij}^s=\left[{o}_1,{o}_2,\dots, {o}_r\right]$ is vector of observations for the *i*-th feature in the *j*-th condition, *r* is the number of the non-missing observations and *s* indicates the set of features with missing data (*w*) or without (*wo*). Furthermore, two models were used for missing data, corresponding to missing at random or completely at random (MAR/MCAR) and missing not at random (MNAR) categories [26]. When there is at least one non-missing observation of a feature in a condition, a MAR/MCAR model is used. We performed MAR/MCAR data imputation by drawing samples from a normal distribution with a mean (${\widehat{\mu}}_{ij}\Big)$ of the non-missing values for that feature and condition (see eq. 2).

2 $$ {\widehat{\mu}}_{ij}=\frac{\sum \limits_{o=1}^r{x}_{ij}^w(r)}{r},{x}_{ij}^w\in \mathbb{R},r= number\ of\ non- missing\ values $$

To estimate of the standard deviation (${\widehat{\sigma}}_j\Big)$ of the normal distribution, we use the median of standard deviations of features without missing data in that condition, ${X}_{\bullet j}^{wo}$ (see eq. 3)

3 $$ {\widehat{\sigma}}_j=\mathrm{m} edian\left(\left[\sigma \left({x}_{1j}^{wo}\right),\sigma \left({x}_{2j}^{wo}\right),\dots, \sigma \left({x}_{Nj}^{wo}\right)\right]\right) $$

as a robust measure of the variation of each feature in the *j*-th condition. Taken together, our imputation model for MAR/MCAR data draws samples from a normal distribution with the mean of the non-missing values in that given feature and condition and with a standard deviation robustly estimated from the features without missing values in that condition (see eq. 4).

4 $$ {x}_{ij}^{MAR/ MCAR}\sim N\left({\widehat{\mu}}_{ij},{\widehat{\sigma}}_j\right) $$

When all observations in a feature and condition are missing, we use a MNAR model. In this case, the imputation model draws samples from a normal distribution:

5 $$ {x}_{ij}^{MNAR}\sim N\left({\widehat{\mu}}_{MNAR},{\widehat{\sigma}}_j\right) $$

with the mean calculated as the lower limit of Tukey fences [25] with *k* value of 1.5 for all observations:

6 $$ {\displaystyle \begin{array}{l}{\widehat{\mu}}_{MNAR}={Q}_1(V)-1.5\left({Q}_3\left(\mathrm{V}\right)-{Q}_1\left(\mathrm{V}\right)\right),{Q}_n(X)=n: th\  quantile\ of\ \mathrm{V},\\ {}V=\left[ vec\left({X}^wo\right); vec\left({X}^w\right)\right]\; is\ the\ vectorized\ form\ of\ the\ data\ matrix.\end{array}} $$

We used multiple imputations in conjunction with binomial testing to decide on statistically significant changes in peptide abundance. Imputation was performed *n* times generating *n* datasets. Relative changes between conditions in peptide abundance were analyzed using *limma’*s [27] function *lmFit* with or without the *method = “robust”* flag, followed by *eBayes* using the default settings [28] and false-discovery rate correction. The logarithmic fold change (LFC) in base two was finnaly calculated for each feature, comparison, and dataset.

We modeled the outcome of the data imputation using a binomial distribution, where each trial of an imputed peptide in a comparative analysis could have two outcomes: significantly changed (having the *p*-value below some set *alpha* level and the LFC above some cut-off level), or insignificantly changed. After counting the outcomes of the *n* imputations, we performed a right-tailed binomial test (using R’s core feature *binom.test* [29]) for observing an outcome (probability of success) significantly higher than 0.5, at a significance level 0.05.

## Results and discussion

PTM quantification by MS has largely been peptide-centric [30], which involves digesting proteins into peptides using protease(s) before enriching samples for a particular modification and detecting with liquid chromatography-tandem mass spectrometry (LC-MS/MS). Although methods available for high-throughput analysis of intact proteoforms are steadily growing [31, 32], a majority of laboratories currently use bottom-up proteomics and peptide-centric quantitation for PTM studies. To develop a robust data analysis workflow for such datasets, we analyzed two distinct experiments featuring: (1) UPS1 standards and (2) enrichment of protein cysteines from the yeast proteome. Both experiments were spiked into Chlamydomonas protein extract at different concentrations to determine our ability to identify changing abundances correctly.

### Performance evaluation

To assess the impact of missing data in the differential analysis, we provide a comparative analysis of multiple imputations using our empirical distribution-based missing data model with Random Forest imputation [20]. We evaluated the performance of the entire data analysis pipeline using combinations of data processing modules to identify the optimal processing pipeline. The performance evaluation used receiver operating characteristics (ROC) curves to compare our pipeline with a *t*-test with and without an LFC cut-off. On the x-axis, we plotted the false positive rate (FPR), against the true positive rate (TPR) on the y-axis (see eq. 7).

7 $$ {\displaystyle \begin{array}{l}\mathrm{FP}\mathrm{R}=\frac{\mathrm{FP}}{\mathrm{FP}+\mathrm{TN}};\mathrm{TPR}=\frac{\mathrm{TP}}{\mathrm{TP}+\mathrm{FN}}\\ {}\mathrm{FP}=\mathrm{False}\ \mathrm{positives},\mathrm{TN}=\mathrm{True}\ \mathrm{negative},\mathrm{TP}=\mathrm{True}\ \mathrm{positives},\mathrm{FN}=\mathrm{False}\ \mathrm{negative}\mathrm{s}.\end{array}} $$

We compared our analysis with an FDR corrected *t*-test using the core R [29] functions (*t.test* with default settings and *p.adjust* with the *method = “fdr”* flag). We also included a hybrid decision method where an LFC cut-off threshold was added to the statistical decision at significance level *alpha* (default 0.05). The *alpha* and LFC cut-off were varied ten times for both the pipeline and the *t*-test (*alpha* between 0.05 and 0.001, and LFC between 0 and 2). The LFC and *alpha* were changed separately or both at the same time. For the *t*-test, a single imputed dataset was used.

Comparison with random forest imputation (which has been shown to display high performance in MS metabolomics data [21]) was performed using the R package missForest [33]. Default settings were used except for *maxiter* which was set to allow for 20 iterations. A single dataset was imputed using random forest and processed through *limma* and using the same hybrid decision method (LFC cut-off and *alpha*).

### Performance analysis using Chlamydomonas total proteome-UPS1 dataset

The *Chlamydomonas*-UPS1 (hereafter referred to as just UPS1) dataset is a global proteomics dataset containing 10,952 features (after filtering) and three different concentrations of spiked-in UPS1: 25, 50, or 100 fmol per 1 μg *Chlamydomonas* lysate (referred to as 25, 50, and 100, respectively) with four replicates each. In each condition, there were 403 confident peptides of UPS1 which were considered as true positives (TP). Since each replicate in each condition was from the same technical Chlamydomonas lysate, they were considered as true negatives (TN).

For the largest difference in concentration (100/25), there was clear discrimination between TP and TN relative to comparisons made between 100/50 and 50/25 (Additional file 3: Figure S1). The comparison with an LFC = 2 had better discrimination between TN and TP and a larger variation of TP (Additional file 3: Figure S1A). At LFC = 1 (100/50 and 50/25), the TP had lower variation, but it was harder to discriminate from TN (Additional file 3: Figure S1B and S1C). There were a total of 1083 (0.82%) missing values in 471 (4.3%) features for which imputation was performed.

**Fig. 2:**
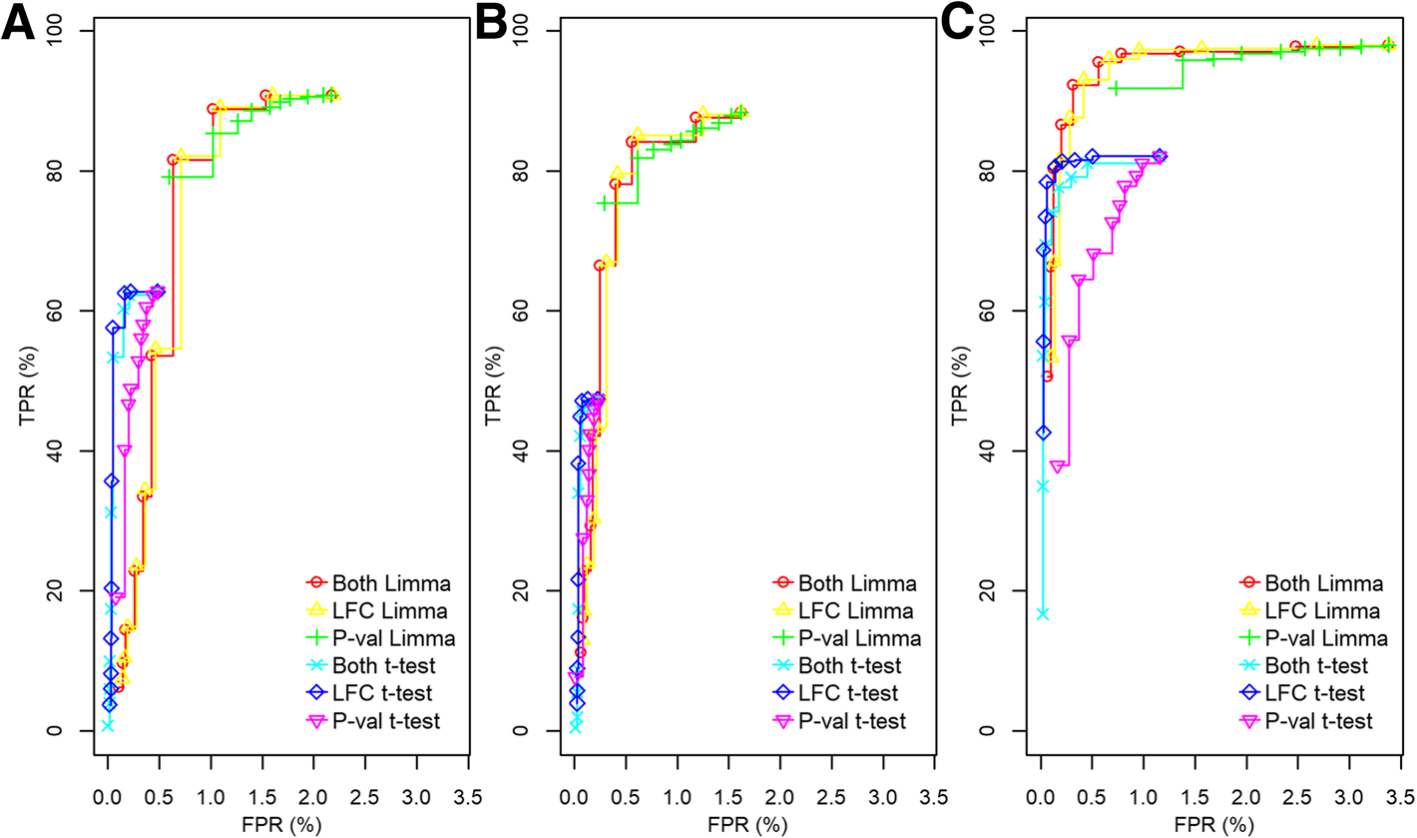
ROC curves of the UPS1 dataset comparing our pipeline with a FDR corrected *t*-test. **a** and **b** show ROC curves of comparisons with a fold change of 2 (LFC of 1), where (**a**) shows comparison between 100 fmol and 50 fmol, and (**b**) shows comparison between 50 fmol and 25 fmol. **c** shows a comparison with a fold change of 4 (LFC of 2) comparing 100 fmol with 25 fmol. Both, LFC, and P-val indicates which parameters were varied (*p*-value and LFC at the same time, LFC only, and p-value only, respectively) when creating the ROC curves. LFC was changed from zero to two and p-value from 0.05 to 0.001, simultaneously or separately. When only LFC was changed the p-value was fixed to 0.05, and when the *p*-value was changed LFC was fixed to zero. Lower LFC cut-off was set to –infinity as there was no TP with a decreasing fold change. “Limma” indicates that our pipeline utilizing *limma* was used with 100 imputations while t-test implies that a FDR corrected *t*-test was used on a single imputed dataset. Y-axis show TPR and x-axis show FPR expressed as a percentage (TPR*100, FPR*100, respectively)

To evaluate the performance of the pipeline, we plotted the receiver operating characteristic (ROC) curves by varying the LFC or *alpha* cut-off, or both simultaneously. *Alpha* was set to 0.05 when changing LFC, while LFC was set to 0 when changing *alpha*. We started by examining the performance of *limma* when using normal or robust regression and found that robust regression outperformed normal regression in all comparisons by achieving a higher TPR when allowing an FPR below 3% (Additional file 4: Figure S2). Accordingly, robust regression was used for all comparisons in the UPS1 dataset. We then compared our pipeline to an FDR corrected *t*-test (hereafter referred to as *t*-test only) with or without an LFC cut-off. The lower LFC cut-off was set to infinity as all TP had a positive LFC. We found that for comparisons in conditions with an LFC of 1 (100/50 and 50/25), our pipeline performed equally well in either, while the *t*-test had reduced performance at the comparison of the lower concentrations (50/25, Fig. 2a and b). When running the comparison with an LFC of 2 (100/25), both *t*-test and our pipeline detected more TP, but with slightly more FP, while the *t*-test was more conservative - missing more TP and fewer FP (Fig. 2c). Notably, the LFC criterion was the most important parameter for both our pipeline and the *t*-test, regardless if simultaneously varying both criteria or only the LFC.

*Performance analysis using redox-enriched Chlamydomonas-yeast dataset.*

The Chlamydomonas-yeast dataset is from an enrichment method for proteins bearing reversibly oxidized cysteine residues [21]. It contained six technical replicates of Chlamydomonas lysate, where three were spiked with 50 ng yeast proteome per 1 μg Chlamydomonas lysate while the other three received 100 ng yeast per 1 μg Chlamydomonas lysate. In total, it contained 2229 peptides with previously oxidized Cys, of which 449 were TP yeast features. Compared to the UPS1 dataset, discrimination between TP and TF in this dataset was more difficult due to increased overlap of TP and TF distributions in compared conditions (see scatterplots in Additional file 3: Figure S1 and Additional file 5: Figure S3). It had a total of 963 (7.2%) missing values in 435 (20%) features for which imputation was performed.

**Fig. 3:**
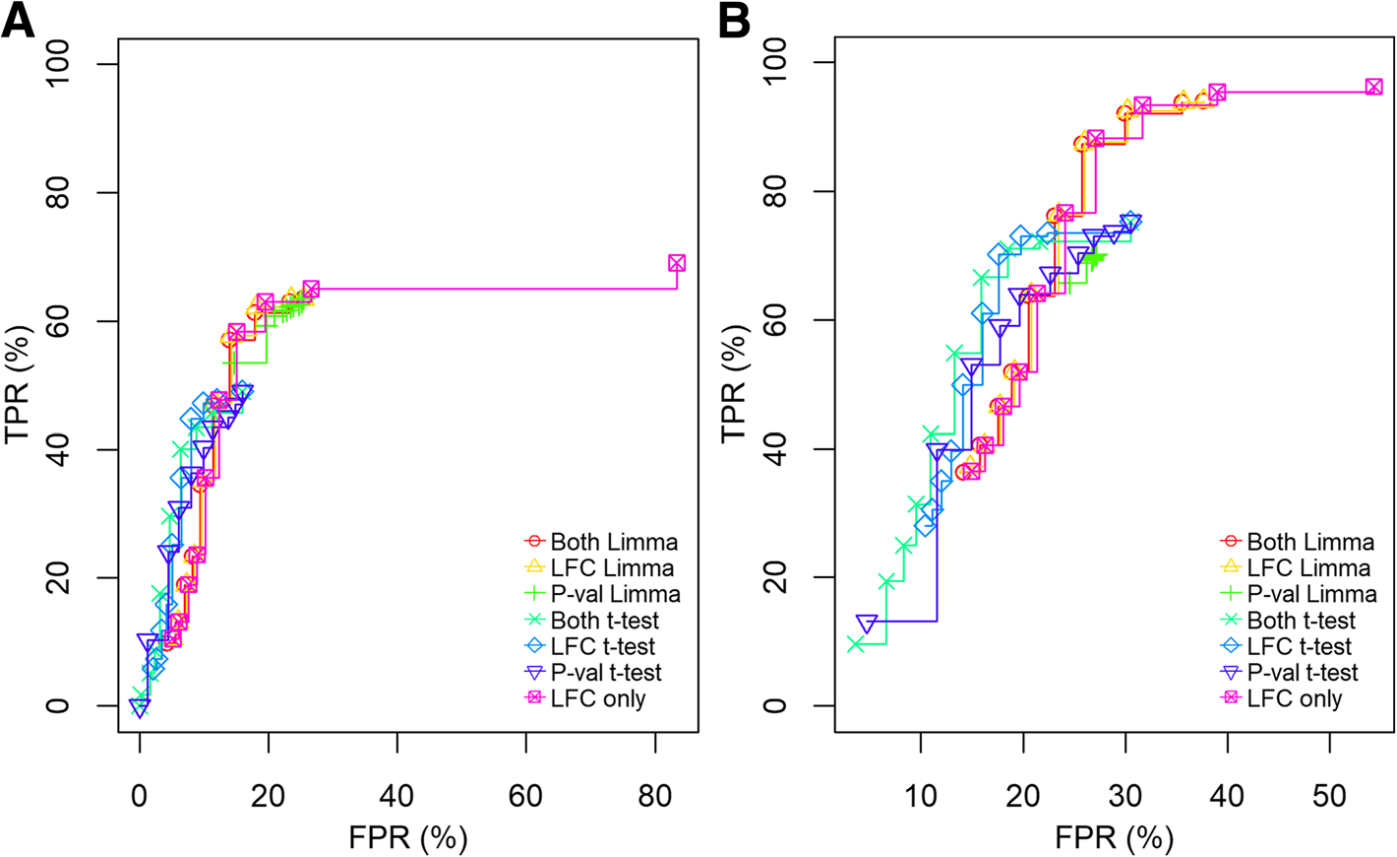
ROC curves of the Yeast dataset comparing our pipeline with a FDR corrected *t*-test, and comparing filtering features with missing values with imputing missing values. **a** shows the performance of limma against FDR corrected *t*-test on the yeast dataset where all features with missing data have been filtered out. **b** compared our pipeline using multiple imputation and limma against *t*-test on a single imputation. Both, LFC, and P-val indicates which parameters were varied (p-value and LFC at the same time, LFC only, and p-value only, respectively) when creating the ROC curves. LFC was changed from zero to two and p-value from 0.05 to 0.001, simultaneously or separately. When only LFC was changed the p-value was fixed to 0.05, and when the p-value was changed LFC was fixed to zero. Lower LFC cut-off was set to –infinity as there was no TP with a decreasing fold change. “Limma” indicates that our pipeline utilizing *limma* was used with 100 imputations while “t-test” indicates that a FDR corrected *t*-test was used on a single imputed dataset. Y-axis show TPR and x-axis show FPR expressed as a percentage (TPR*100, FPR*100, respectively)

Similar to the UPS1 analysis, performance was evaluated with ROC curves for the yeast dataset, and started by comparing whether *limma* ran best with robust or normal regression. We found that normal regression improved performance over robust regression and that robust regression had, on average, a lower TPR and a higher FPR (Additional file 6: Figure S4). We decided to use *limma* with normal regression for the yeast dataset. Comparing our pipeline with a *t*-test, we first varied either *alpha* or LFC cut-off, or both at the same time, using values identical to the UPS1 dataset. We found that the *t*-test had a higher TPR and lower FPR at more stringent settings-independent if the criterions were changed individually or at the same time - but it again failed to reach as high TPR as our pipeline (Fig. 3a). At less stringent settings, our pipeline could almost identify all TP at the expense of a small increase in FP. We then performed imputation on the missing values. For the *t*-test, we generated one imputed dataset, while for our pipeline we performed 100 multiple imputations. We found that both the *t*-test and our pipeline improved in TP, but both picked up more FP (Fig. 3b). We found that the dynamics in the TPR and FPR were similar between a *t*-test and our pipeline regardless if the data was filtered or imputed. Importantly, we found that performing imputation instead of filtering out features with missing values lead to a drastic improvement in TP for both *t*-test and our pipeline. We also found that the parameter with the highest impact was LFC, which was consistent with the results of the UPS1 dataset.

**Fig. 4:**
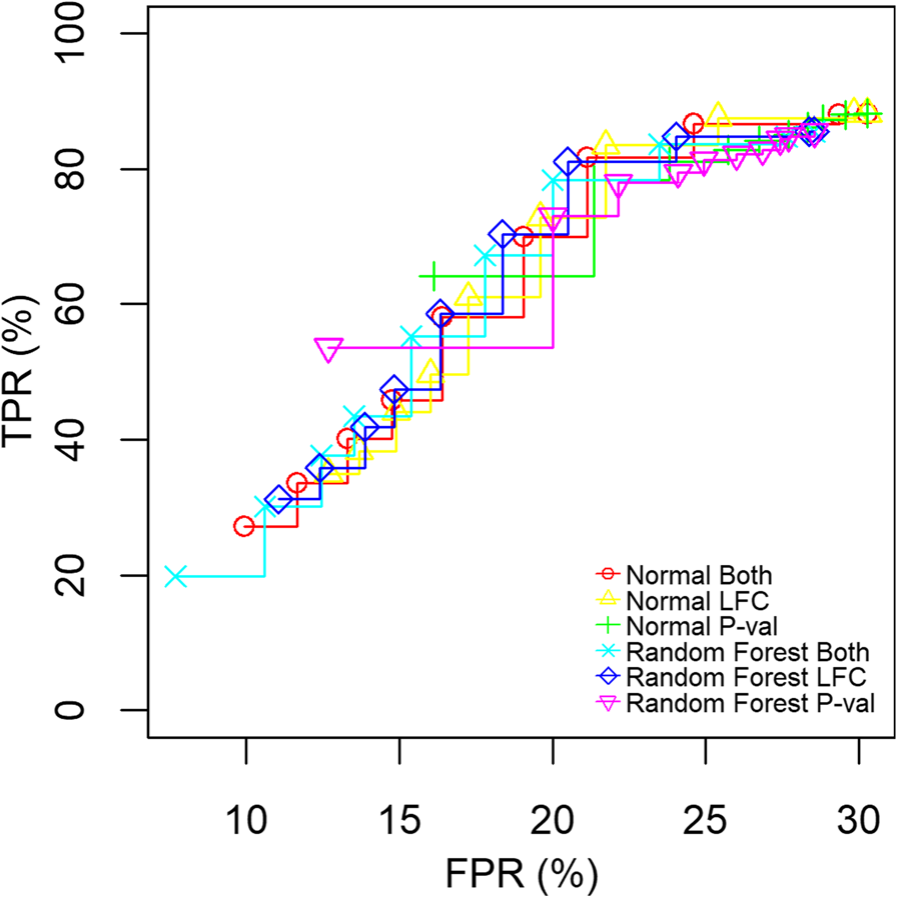
ROC curves comparing the performance of our pipeline with a method using random forest imputation and linear regression analysis. A single imputation using a random forest algorithm from the R package missForest was compared to our pipeline running 100 imputations. We applied a criterion of either a LFC cut-off, a *p*-value, or both at the same time. Both, LFC, and P-val indicates which parameters were varied (p-value and LFC at the same time, LFC only, and p-value only, respectively) when creating the ROC curves. LFC was changed from zero to two and p-value from 0.05 to 0.001, simultaneously or separately. When only LFC was changed the p-value was fixed to 0.05, and when the p-value was changed LFC was fixed to zero. “Random Forest” indicates that the missForest packages were used for the imputation and “Normal” indicates that our pipeline was used with *limma* with normal regression. Y-axis show TPR and x-axis show FPR expressed as a percentage (TPR*100, FPR*100, respectively)

We compared our imputation method with random forest imputation (using the *missForest* package [33]). We found that our multiple-imputation method had a slightly better performance versus random forest imputation (Fig. 4) regarding the maximum TPR achievable and when allowing a large FPR (larger than 20%) while performing comparable at lower FPRs. While Random Forest missing data algorithms perform better for high correlation data, their performance generally degrades for missing not at random data [34]. Our multiple imputation method show increased robustness as a result of binomial testing of the outcome of linear regression analysis.

Computationally, our method is inexpensive since it samples normal distributions to impute missing data. Improved missing data models can be developed from information on the missing-ness mechanisms related to the PTM quantification protocols. Stochastic sampling methods [35] can be used for left-censored missing value imputation for MNAR in combination with bootstrapping and other statistical resampling methods for MAR/MCAR imputation when the percentage of missing data is expected to be large.

## Conclusion

PTM detection and analysis in comparative assays introduce new challenges in data imputation. Enrichment protocols may exhibit high variation among technical replicates and structure dependent-bias, leading to an increase in the percentage of missing data. Here we set up a benchmark dataset to analyze the performance of a pipeline developed in the R programming language including data imputation, *limma* analysis, and multiple imputation binomial testing, in comparison to a traditional pipeline including statistical testing using *t*-test and FDR correction for multiple testing. Robust regression methods were expected to outperform typical statistical tests used in MS data analysis [36]. Here we conducted a performance evaluation of the pipelines for total proteome quantitation and differential analysis of redox proteome. Our results indicate that a significant improvement in performance can be obtained when using a robust estimation of missing data distribution parameters combined with linear modeling and binomial testing.

We also compared our imputation method with random forest imputation and found that our method had a slightly better performance at high FPRs, while performing similarly at lower FPRs. Given that our multiple-imputation method can use a learning strategy to optimize performance over the entire pipeline, further gains can be obtained when compared with a bootstrap imputation that is sampling only the input data.

## Additional files

Additional file 1: File containing the implementation of Progenesis LFQ workflow for UPS1 dataset. (PDF 206 kb)

Additional file 2: File containing the implementation of Progenesis LFQ workflow for *Chlamydomonas-yeast* dataset. (PDF 208 kb)

Additional file 3: **Figure S1.** Scatterplots of the UPS1 dataset showing the position of the true positives and the true negatives. A shows a comparison between 100 fmol (y-axis) and 25 fmol (x-axis; fold change of 4) spiked-in UPS1 protein. B shows a comparison between 100 fmol (y-axis) and 50 fmol (x-axis) and C shows comparisons of between 50 fmol (y-axis) and 25 fmol (x-axis; both comparisons having a fold change of 2). Each circle represents the mean of all replicates after running our imputation one time. True negatives (TN) was marked in red and true positives (TP) was marked in blue. All TN were Chlamydomonas peptides. (TIFF 166 kb)

Additional file 4: **Figure S2.** ROC curves comparison of using limma with or without robust regression in our pipeline with the UPS1 dataset. The **A-B** show ROC curves of comparisons with a fold change of 2 (LFC of 1), **A** shows comparison between 100 fmol and 50 fmol, and **B** shows comparison between 50 fmol and 25 fmol. **C** shows a comparison with a fold change of 4 (LFC of 2) comparing the lowest and highest spike-in concentrations. Both, LFC, and P-val indicate which parameters were varied (p-value and LFC at the same time, LFC only, and p-value only, respectively) when creating the ROC curves. LFC was changed from zero to two and p-value from 0.05 to 0.001, simultaneously or separately. When only LFC was changed the p-value was fixed to 0.05, and when the p-value was changed LFC was fixed to zero. Lower LFC cut-off was set to –infinity as there was no TP with a decreasing fold change. “Robust” indicates *limma* with robust regression and “Normal” indicates *limma* using normal regression. Y-axis show TPR and x-axis show FPR expressed as a percentage (TPR*100, FPR*100, respectively). (TIFF 362 kb)

Additional file 5: **Figure S3.** Scatterplot of the yeast dataset showing the position of the true positives and the true negatives. Comparison between 100 ng (y-axis) and 50 ng (x-axis) yeast protein spike-in enriched for reversibly oxidized cysteines, in a background of *Chlamydomonas reinhardtii* lysate. Each dot represents the mean of all replicates after running our imputation one time. True negatives (TN) was marked in red and true positives (TP) was marked in blue. (TIFF 172 kb)

Additional file 6: **Figure S4.** ROC curves comparison of using limma with or without robust regression in our pipeline with the yeast dataset. Both, LFC, and P-val indicates which parameters were varied (p-value and LFC at the same time, LFC only, and p-value only, respectively) when creating the ROC curves. LFC was changed from zero to two and p-value from 0.05 to 0.001, simultaneously or separately. When only LFC was changed the p-value was fixed to 0.05, and when the p-value was changed LFC was fixed to zero. Lower LFC cut-off was set to –infinity as there was no TP with a decreasing fold change. “Robust” indicates *limma* with robust regression and “Normal” indicates *limma* using normal regression. Y-axis show TPR and x-axis show FPR expressed as a percentage (TPR*100, FPR*100, respectively). (TIFF 39 kb)

Additional file 7: Archive containing the R (version 3.4.4) implementation of the computational pipeline. (RAR 15 kb)

Additional file 8: File containing a tutorial on using the computational pipeline. (PDF 1543 kb)

## Abbreviations

FPR: False positive rate
LFC: Logarithmic fold change
*Limma*: Linear Models for Microarray Data
MAR: Missing at random
MCAR: Missing completely at random
MNAR: Missing not at random
PTM: Post-translational modification
ROC: Receiver operating characteristics
TPR: True positive rate
UPS1: Universal Proteomics Standard Set 1
