# Supplementary material for: Evaluation of linear models and missing value imputation for the analysis of peptide-centric proteomics
Source: BMC Bioinformatics. 2019 Mar 14;20(Suppl 2):102. doi: 10.1186/s12859-019-2619-6 (PMC6419331; doi:10.1186/s12859-019-2619-6)
Supplement: Supplementary file 1 — File containing the implementation of Progenesis LFQ workflow for UPS1 dataset. (PDF 206 kb) [file 12859_2019_2619_MOESM1_ESM.pdf]

Progenesis LFQ global workflow

Contents

Load functions

```
source("./EWM_ProgenesisLFQ_redox_v2.R")
```

Required packages

```
# Load required packages

#install.packages("tidyverse")
require(tidyverse)

#source("https://bioconductor.org/biocLite.R")
#biocLite("Biostrings")
require(Biostrings)
```

Data input

Filename	Type
Peptide measurements	.csv
Protein measurements	.csv
FASTA database	.fasta

Peptide measurements

```
pepm <- read.csv("./20180502_WOS52_Cr_UPS_pepm.csv", skip = 2, stringsAsFactors = FALSE)
```

```
##  [1] "X."                      "Retention.time..min."
##  [3] "Charge"                  "m.z"
##  [5] "Measured.mass"           "Mass.error..u."
##  [7] "Mass.error..ppm."        "Score"
##  [9] "Sequence"                "Modifications"
## [11] "Accession"               "Description"
## [13] "Use.in.quantitation"     "Max.fold.change"
## [15] "Highest.mean.condition" "Lowest.mean.condition"
## [17] "Anova"                   "Maximum.CV"
## [19] "X20141222_WOS521"        "X20141222_WOS526"
## [21] "X20141222_WOS5211"       "X20141222_WOS5216"
## [23] "X20141222_WOS522"        "X20141222_WOS527"
## [25] "X20141222_WOS5212"       "X20141222_WOS5217"
## [27] "X20141222_WOS523"        "X20141222_WOS528"
## [29] "X20141222_WOS5213"       "X20141222_WOS5218"
## [31] "X20141222_WOS521.1"      "X20141222_WOS526.1"
```

```
## [33] "X20141222_WOS5211.1"    "X20141222_WOS5216.1"
## [35] "X20141222_WOS522.1"     "X20141222_WOS527.1"
## [37] "X20141222_WOS5212.1"    "X20141222_WOS5217.1"
## [39] "X20141222_WOS523.1"     "X20141222_WOS528.1"
## [41] "X20141222_WOS5213.1"    "X20141222_WOS5218.1"
## [43] "X20141222_WOS521.2"     "X20141222_WOS526.2"
## [45] "X20141222_WOS5211.2"    "X20141222_WOS5216.2"
## [47] "X20141222_WOS522.2"     "X20141222_WOS527.2"
## [49] "X20141222_WOS5212.2"    "X20141222_WOS5217.2"
## [51] "X20141222_WOS523.2"     "X20141222_WOS528.2"
## [53] "X20141222_WOS5213.2"    "X20141222_WOS5218.2"
```

Protein measurements

```
protm <- read.csv("./20180502_WOS52_Cr_UPS_protm.csv", skip = 2, stringsAsFactors = FALSE)
```

```
##  [1] "Accession"              "Peptide.count"
##  [3] "Unique.peptides"        "Confidence.score"
##  [5] "Anova..p."              "Max.fold.change"
##  [7] "Highest.mean.condition" "Lowest.mean.condition"
##  [9] "Description"            "X20141222_WOS521"
## [11] "X20141222_WOS526"       "X20141222_WOS5211"
## [13] "X20141222_WOS5216"      "X20141222_WOS522"
## [15] "X20141222_WOS527"       "X20141222_WOS5212"
## [17] "X20141222_WOS5217"      "X20141222_WOS523"
## [19] "X20141222_WOS528"       "X20141222_WOS5213"
## [21] "X20141222_WOS5218"      "X20141222_WOS521.1"
## [23] "X20141222_WOS526.1"     "X20141222_WOS5211.1"
## [25] "X20141222_WOS5216.1"    "X20141222_WOS522.1"
## [27] "X20141222_WOS527.1"     "X20141222_WOS5212.1"
## [29] "X20141222_WOS5217.1"    "X20141222_WOS523.1"
## [31] "X20141222_WOS528.1"     "X20141222_WOS5213.1"
## [33] "X20141222_WOS5218.1"
```

FASTA database

```
database <- readAAStringSet("./20180502_Cr_mt_chl_UPS.fasta")

# Split header by first space
names(database) <- sapply(strsplit(names(database), " "), head, 1)
```

```
##   A AAStringSet instance of length 19651
##         width seq                                          names
##     [1]   155 MDATSKADLPDYAADNRLP...SAATPYTTTQSPPSTTLS Cre38.g759997.t1....
##     [2]   575 MGGAKRRRGATAEDPETPG...DRGKYSADGVTVCGRGLH Cre08.g363350.t1....
##     [3]   664 MEAPTQRICRVCARQYARY...EASGGGGPNWRPGDVRVQ Cre08.g379050.t1....
##     [4]  3975 MEPSITRLTASARDRVGDR...QLAPPGAAGGPQLTGTAV Cre08.g364751.t1....
##     [5]   318 MQVSRAAVCARSSFVRVQA...ARAKSVFQEIAASAPKQA Cre08.g372000.t1....
##     ...   ... ...
## [19647]   109 SLGSLTIAEPAMIAECKTR...EDHLACKCETVAAARPVT P01127ups|PDGFB_H...
## [19648]   146 VHLTPEEKSAVTALWGKVN...AYQKVVAGVANALAHKYH P68871ups|HBB_HUM...
## [19649]   158 MSGIALSRLAQERKAWRKD...RVEYEKRVRAQAKKFAPS P63279ups|UBC9_HU...
```

```
## [19650]   147 MGSSHHHHHHSSGLVPRGS...EASKEKEEVAEEAQSGGD O76070ups|SYUG_HU...
## [19651]   143 QDPYVKEAENLKKYFNAGH...TGKRKRSQMLFRGRRASQ P01579ups|IFNG_HU...
```

Define conditions/samples

```r
A <- 19:22
B <- 23:26
C <- 27:30

group <- list(A, B, C)
```

```
## [[1]]
## [1] 19 20 21 22
##
## [[2]]
## [1] 23 24 25 26
##
## [[3]]
## [1] 27 28 29 30
```

Filter score & contaminants

- Remove peptide identifications with Mascot score < 13 and any cRAP matches

```r
pepm <- pepm %>%
  filter_score() %>%
  filter_contaminants()
```

```
## Observations: 13,452
## Variables: 54
## $ X.                      <int> 10, 26, 22333, 28718, 55, 58, 71, 72, 7...
## $ Retention.time..min.    <dbl> 55.36642, 60.87572, 63.36590, 100.43320...
## $ Charge                  <int> 2, 2, 2, 2, 2, 2, 2, 2, 3, 2, 2, 3, 2, ...
## $ m.z                     <dbl> 435.2949, 725.3925, 725.3840, 725.3931,...
## $ Measured.mass           <dbl> 868.5753, 1448.7704, 1448.7535, 1448.77...
## $ Mass.error..u.          <dbl> 0.0007034599, 0.0007219489, -0.01618253...
## $ Mass.error..ppm.        <dbl> 0.8099016, 0.4983186, -11.1698449, 1.33...
## $ Score                   <dbl> 34.40, 67.73, 30.91, 47.01, 60.17, 42.8...
## $ Sequence                <chr> "LINLLGVK", "IAFLDGNTAVMLGK", "IAFLDGNT...
## $ Modifications           <chr> "", "", "", "", "", "", "", "", "", "",...
## $ Accession               <chr> "Cre06.g263450.t1.2|PACid:30779125|", "...
## $ Description             <chr> "GTP binding Elongation factor Tu famil...
## $ Use.in.quantitation     <chr> "True", "True", "False", "False", "Fals...
## $ Max.fold.change         <dbl> 1.103082, 1.037959, 1.141527, 2.099122,...
## $ Highest.mean.condition <chr> "25 fmol", "25 fmol", "100 fmol", "25 f...
## $ Lowest.mean.condition   <chr> "100 fmol", "100 fmol", "25 fmol", "100...
## $ Anova                   <dbl> 2.321945e-02, 5.441127e-01, 8.549124e-0...
## $ Maximum.CV              <dbl> 5.323264, 6.139954, 40.154356, 16.43890...
## $ X20141222_WOS521        <dbl> 56721.99633, 63392.30014, 255.92515, 99...
## $ X20141222_WOS526        <dbl> 55625.44316, 57604.48940, 270.10856, 10...
## $ X20141222_WOS5211       <dbl> 53959.6955, 58688.1055, 192.0700, 109.9...
## $ X20141222_WOS5216       <dbl> 51366.28868, 59007.60799, 91.64452, 141...
## $ X20141222_WOS522        <dbl> 50831.13972, 54126.35674, 276.09234, 69...
```

```
## $ X20141222_WOS527       <dbl> 50058.77654, 56129.15081, 249.94143, 83...
## $ X20141222_WOS5212      <dbl> 52380.81606, 59494.14321, 212.84666, 57...
## $ X20141222_WOS5217      <dbl> 49549.84148, 62156.89246, 103.07629, 79...
## $ X20141222_WOS523       <dbl> 46915.24682, 55679.40827, 281.06073, 56...
## $ X20141222_WOS528       <dbl> 50131.97016, 57882.19495, 268.57499, 53...
## $ X20141222_WOS5213      <dbl> 47603.20356, 55799.74197, 243.72299, 53...
## $ X20141222_WOS5218      <dbl> 52681.663434, 60602.081107, 130.990881,...
## $ X20141222_WOS521.1     <dbl> 51371.59150, 57412.70685, 231.78454, 90...
## $ X20141222_WOS526.1     <dbl> 53033.62581, 54920.46018, 257.52310, 10...
## $ X20141222_WOS5211.1    <dbl> 53959.6955, 58688.1055, 192.0700, 109.9...
## $ X20141222_WOS5216.1    <dbl> 51961.47761, 59691.33805, 92.70641, 142...
## $ X20141222_WOS522.1     <dbl> 55010.8030, 58576.9740, 298.7944, 75.35...
## $ X20141222_WOS527.1     <dbl> 50026.95398, 56093.46929, 249.78254, 83...
## $ X20141222_WOS5212.1    <dbl> 51890.48507, 58937.22516, 210.85423, 56...
## $ X20141222_WOS5217.1    <dbl> 49740.15656, 62395.62972, 103.47220, 79...
## $ X20141222_WOS523.1     <dbl> 44565.47142, 52890.67513, 266.98365, 54...
## $ X20141222_WOS528.1     <dbl> 49908.22530, 57623.85992, 267.37631, 53...
## $ X20141222_WOS5213.1    <dbl> 45616.75253, 53471.25467, 233.55258, 51...
## $ X20141222_WOS5218.1    <dbl> 51065.523974, 58742.963375, 126.972414,...
## $ X20141222_WOS521.2     <int> 6, 6, 2, 0, 4, 4, 4, 3, 4, 1, 3, 3, 0, ...
## $ X20141222_WOS526.2     <int> 7, 7, 4, 0, 3, 4, 5, 3, 4, 1, 3, 4, 0, ...
## $ X20141222_WOS5211.2    <int> 6, 7, 1, 0, 4, 5, 4, 3, 4, 1, 3, 3, 0, ...
## $ X20141222_WOS5216.2    <int> 6, 7, 1, 0, 4, 5, 3, 3, 3, 1, 3, 4, 0, ...
## $ X20141222_WOS522.2     <int> 5, 4, 2, 2, 5, 4, 3, 3, 4, 0, 3, 4, 0, ...
## $ X20141222_WOS527.2     <int> 5, 6, 1, 1, 4, 6, 4, 3, 3, 1, 3, 4, 0, ...
## $ X20141222_WOS5212.2    <int> 5, 5, 2, 0, 3, 5, 4, 3, 3, 1, 3, 3, 0, ...
## $ X20141222_WOS5217.2    <int> 5, 7, 2, 0, 4, 5, 4, 3, 4, 1, 3, 3, 0, ...
## $ X20141222_WOS523.2     <int> 5, 6, 2, 0, 4, 4, 3, 7, 3, 1, 3, 3, 0, ...
## $ X20141222_WOS528.2     <int> 6, 7, 4, 3, 3, 5, 3, 3, 3, 1, 3, 2, 3, ...
## $ X20141222_WOS5213.2    <int> 6, 6, 4, 1, 3, 4, 3, 3, 3, 1, 3, 4, 0, ...
## $ X20141222_WOS5218.2    <int> 7, 6, 2, 1, 3, 5, 3, 3, 3, 1, 3, 3, 0, ...
```

Summarize duplicate features

- There were instances of rows with duplicated peak features and differing peptide identifications:
  - Some features were matched with peptides having identical sequence, modifications, and score, but alternate protein accessions. These groups were reduced to satisfy the principle of parsimony and represented by the protein accession with the highest number of unique peptides, else the protein with the largest confidence score assigned by Progenesis.
  - Some features were also duplicated with differing peptide identifications and were reduced to a single peptide with the highest Mascot ion score.

```
pepm <- pepm %>%
  protm_map(protm) %>%
  pepm_reduce()
```

```
## Observations: 12,792
## Variables: 3
## $ Peptide.count    <int> 6, 48, 48, 11, 32, 59, 21, 48, 48, 15, 48, 48...
## $ Unique.peptides  <int> 6, 47, 47, 6, 29, 58, 20, 47, 47, 10, 47, 47,...
## $ Confidence.score <dbl> 500.83, 2910.63, 2910.63, 663.93, 1605.89, 42...
```

Filter for unique peptides

- An identifier was made by joining the protein accession of each feature with the peptide sequence.

```
pepm <- pepm %>%
  get_identifier()
```

```
## Observations: 12,792
## Variables: 1
## $ Identifier <chr> "Cre03.g182551.t1.2|PACid:30787991|--LGADSGALEFVPK"...
```

Summarize duplicate identifiers

- The dataset was then reduced to unique identifiers by summing the abundance of all contributing features (*i.e.*, peptide charge states, missed cleavages, and combinations of additional variable modifications).
- Each identifier group was represented by the peptide with the highest Mascot score in the final dataset.

```
pepm <- pepm %>%
  identifier_reduce(group)
```

```
## Observations: 10,599
## Variables: 58
## $ X.                      <int> 8219, 6089, 10453, 6835, 36039, 15494, ...
## $ Retention.time..min.    <dbl> 64.61362, 43.22110, 66.59455, 28.56493,...
## $ Charge                  <int> 2, 3, 3, 2, 3, 2, 3, 2, 3, 2, 3, 3, 2, ...
## $ m.z                     <dbl> 751.4402, 468.9241, 776.7716, 499.2639,...
## $ Measured.mass           <dbl> 1500.8658, 1403.7504, 2327.2931, 996.51...
## $ Mass.error..u.          <dbl> -6.326263e-04, -1.736175e-03, 4.059479e...
## $ Mass.error..ppm.        <dbl> -0.42150742, -1.23681040, 1.74429514, 0...
## $ Score                   <dbl> 86.33, 38.68, 39.88, 44.78, 42.39, 46.4...
## $ Sequence                <chr> "AVLLFATGSGISPLR", "GFALDRLPASTTR", "VV...
## $ Modifications           <chr> "", "", "", "", "", "", "", "", "", "",...
## $ Accession               <chr> "Cre01.g000350.t1.1|PACid:30788481|", "...
## $ Description             <chr> "FAD/NAD(P)-binding oxidoreductase", "F...
## $ Use.in.quantitation     <chr> "True", "True", "True", "False", "True"...
## $ Max.fold.change         <dbl> 1.093262, 1.188561, 1.075179, 1.109310,...
## $ Highest.mean.condition  <chr> "50 fmol", "50 fmol", "25 fmol", "25 fm...
## $ Lowest.mean.condition   <chr> "25 fmol", "100 fmol", "100 fmol", "50 ...
## $ Anova                   <dbl> 0.024735469, 0.008415748, 0.425207932, ...
## $ Maximum.CV              <dbl> 5.864683, 8.353106, 9.780676, 10.326684...
## $ X20141222_WOS521        <dbl> 695.233106, 736.010277, 680.923568, 491...
## $ X20141222_WOS526        <dbl> 673.702805, 636.239273, 726.962918, 447...
## $ X20141222_WOS5211       <dbl> 688.88641, 656.68287, 637.84382, 463.34...
## $ X20141222_WOS5216       <dbl> 694.08555, 607.61558, 646.63941, 443.72...
## $ X20141222_WOS522        <dbl> 740.53020, 793.16318, 732.25942, 372.36...
## $ X20141222_WOS527        <dbl> 738.76202, 782.66764, 725.61366, 408.25...
## $ X20141222_WOS5212       <dbl> 732.79959, 694.18539, 653.83181, 408.18...
## $ X20141222_WOS5217       <dbl> 796.464769, 734.341036, 661.200365, 475...
## $ X20141222_WOS523        <dbl> 644.41560, 653.91101, 628.36948, 413.27...
## $ X20141222_WOS528        <dbl> 674.76970, 641.56616, 693.96853, 388.88...
## $ X20141222_WOS5213       <dbl> 714.50165, 619.09480, 607.19168, 476.20...
## $ X20141222_WOS5218       <dbl> 735.33006, 613.15538, 683.97484, 433.07...
## $ X20141222_WOS521.1      <dbl> 629.653987, 666.584777, 553.150581, 444...
## $ X20141222_WOS526.1      <dbl> 642.312230, 606.594278, 636.105690, 426...
```

```
## $ X20141222_WOS5211.1    <dbl> 688.88641, 656.68287, 555.66456, 463.34...
## $ X20141222_WOS5216.1    <dbl> 702.12803, 614.65612, 545.53043, 448.86...
## $ X20141222_WOS522.1     <dbl> 801.42136, 858.38216, 672.22183, 402.97...
## $ X20141222_WOS527.1     <dbl> 738.29238, 782.17009, 617.64475, 407.99...
## $ X20141222_WOS5212.1    <dbl> 725.93993, 687.68720, 546.73824, 404.36...
## $ X20141222_WOS5217.1    <dbl> 799.523896, 737.161553, 540.521835, 477...
## $ X20141222_WOS523.1     <dbl> 612.13969, 621.15953, 541.51023, 392.57...
## $ X20141222_WOS528.1     <dbl> 671.75812, 638.70277, 569.99602, 387.14...
## $ X20141222_WOS5213.1    <dbl> 684.68596, 593.26038, 500.41537, 456.33...
## $ X20141222_WOS5218.1    <dbl> 712.77200, 594.34533, 525.52967, 419.78...
## $ X20141222_WOS521.2     <int> 2, 1, 1, 1, 0, 0, 0, 0, 1, 1, 1, 0, 2, ...
## $ X20141222_WOS526.2     <int> 2, 1, 2, 1, 0, 0, 1, 1, 1, 1, 1, 0, 1, ...
## $ X20141222_WOS5211.2    <int> 2, 1, 1, 1, 0, 0, 0, 1, 1, 1, 1, 1, 2, ...
## $ X20141222_WOS5216.2    <int> 2, 1, 1, 1, 1, 0, 1, 1, 1, 1, 1, 0, 2, ...
## $ X20141222_WOS522.2     <int> 2, 1, 1, 1, 0, 0, 0, 0, 2, 2, 1, 0, 1, ...
## $ X20141222_WOS527.2     <int> 2, 1, 1, 1, 1, 0, 0, 0, 1, 2, 1, 1, 2, ...
## $ X20141222_WOS5212.2    <int> 2, 1, 1, 1, 0, 0, 0, 1, 1, 1, 1, 1, 1, ...
## $ X20141222_WOS5217.2    <int> 2, 1, 1, 1, 0, 1, 1, 0, 1, 2, 1, 1, 1, ...
## $ X20141222_WOS523.2     <int> 2, 1, 2, 1, 0, 0, 1, 0, 1, 1, 1, 1, 1, ...
## $ X20141222_WOS528.2     <int> 2, 1, 1, 1, 0, 0, 1, 0, 1, 1, 1, 0, 2, ...
## $ X20141222_WOS5213.2    <int> 2, 1, 2, 1, 0, 0, 1, 1, 1, 1, 1, 1, 2, ...
## $ X20141222_WOS5218.2    <int> 2, 1, 1, 1, 0, 0, 1, 1, 1, 1, 1, 1, 1, ...
## $ Peptide.count          <int> 5, 5, 5, 5, 3, 3, 3, 15, 15, 15, 15, 15...
## $ Unique.peptides        <int> 4, 4, 4, 4, 3, 3, 3, 14, 14, 14, 14, 14...
## $ Confidence.score       <dbl> 253.88, 253.88, 253.88, 253.88, 117.75,...
## $ Identifier             <chr> "Cre01.g000350.t1.1|PACid:30788481|--AV...
```

Select necessary variables

- Reduce variables to simplify in downstream processing
- Replace variable names with simple 'Letter-Number' format

```
pepm <- pepm %>%
  lfq_simplify(group)

colnames(pepm) <- get_design(group) %>%
  c("Identifier", .)
```

```
## Observations: 10,599
## Variables: 13
## $ Identifier <chr> "Cre01.g000350.t1.1|PACid:30788481|--AVLLFATGSGISPL...
## $ `A-1`      <dbl> 695.233106, 736.010277, 680.923568, 491.323735, 7.8...
## $ `A-2`      <dbl> 673.702805, 636.239273, 726.962918, 447.562981, 1.1...
## $ `A-3`      <dbl> 688.88641, 656.68287, 637.84382, 463.34129, 0.00000...
## $ `A-4`      <dbl> 694.08555, 607.61558, 646.63941, 443.72045, 26.9077...
## $ `B-1`      <dbl> 740.53020, 793.16318, 732.25942, 372.36120, 0.00000...
## $ `B-2`      <dbl> 738.76202, 782.66764, 725.61366, 408.25301, 33.4399...
## $ `B-3`      <dbl> 732.79959, 694.18539, 653.83181, 408.18144, 0.00000...
## $ `B-4`      <dbl> 796.464769, 734.341036, 661.200365, 475.256086, 0.0...
## $ `C-1`      <dbl> 644.41560, 653.91101, 628.36948, 413.27051, 0.00000...
## $ `C-2`      <dbl> 674.76970, 641.56616, 693.96853, 388.88483, 0.00000...
## $ `C-3`      <dbl> 714.50165, 619.09480, 607.19168, 476.20902, 0.00000...
## $ `C-4`      <dbl> 735.33006, 613.15538, 683.97484, 433.07009, 0.00000...
```
