# Supplementary material for: Evaluation of linear models and missing value imputation for the analysis of peptide-centric proteomics
Source: BMC Bioinformatics. 2019 Mar 14;20(Suppl 2):102. doi: 10.1186/s12859-019-2619-6 (PMC6419331; doi:10.1186/s12859-019-2619-6)
Supplement: Supplementary file 2 — File containing the implementation of Progenesis LFQ workflow for Chlamydomonas-yeast dataset. (PDF 208 kb) [file 12859_2019_2619_MOESM2_ESM.pdf]

Progenesis LFQ redox workflow

Contents

Load functions

```
source("./EWM_ProgenesisLFQ_redox_v2.R")
```

Required packages

```
# Load required packages

#install.packages("tidyverse")
require(tidyverse)

#source("https://bioconductor.org/biocLite.R")
#biocLite("Biostrings")
require(Biostrings)
```

Data input

Filename	Type
Peptide measurements	.csv
Protein measurements	.csv
FASTA database	.fasta

Peptide measurements

```
pepm <- read.csv("./20180502_EWM47_Cr_yeast_pepm.csv", skip = 2, stringsAsFactors = FALSE)
```

```
##  [1] "X."
##  [2] "Retention.time..min."
##  [3] "Charge"
##  [4] "m.z"
##  [5] "Measured.mass"
##  [6] "Mass.error..u."
##  [7] "Mass.error..ppm."
##  [8] "Score"
##  [9] "Sequence"
## [10] "Modifications"
## [11] "Accession"
## [12] "Description"
## [13] "Use.in.quantitation"
## [14] "Max.fold.change"
## [15] "Highest.mean.condition"
## [16] "Lowest.mean.condition"
```

```
## [17] "Anova"
## [18] "Maximum.CV"
## [19] "X20161007_EWM47_ER_50ng_1_5.35_7uL"
## [20] "X20161007_EWM47_ER_50ng_2_5.35_7uL"
## [21] "X20161007_EWM47_ER_50ng_3_5.35_7uL"
## [22] "X20161007_EWM47_ER_100ng_1_5.35_7uL"
## [23] "X20161007_EWM47_ER_100ng_2_5.35_7uL"
## [24] "X20161007_EWM47_ER_100ng_3_5.35_7uL"
## [25] "X20161007_EWM47_ER_50ng_1_5.35_7uL.1"
## [26] "X20161007_EWM47_ER_50ng_2_5.35_7uL.1"
## [27] "X20161007_EWM47_ER_50ng_3_5.35_7uL.1"
## [28] "X20161007_EWM47_ER_100ng_1_5.35_7uL.1"
## [29] "X20161007_EWM47_ER_100ng_2_5.35_7uL.1"
## [30] "X20161007_EWM47_ER_100ng_3_5.35_7uL.1"
## [31] "X20161007_EWM47_ER_50ng_1_5.35_7uL.2"
## [32] "X20161007_EWM47_ER_50ng_2_5.35_7uL.2"
## [33] "X20161007_EWM47_ER_50ng_3_5.35_7uL.2"
## [34] "X20161007_EWM47_ER_100ng_1_5.35_7uL.2"
## [35] "X20161007_EWM47_ER_100ng_2_5.35_7uL.2"
## [36] "X20161007_EWM47_ER_100ng_3_5.35_7uL.2"
```

Protein measurements

```
protm <- read.csv("./20180502_EWM47_Cr_yeast_protm.csv", skip = 2, stringsAsFactors = FALSE)
```

```
##  [1] "Accession"
##  [2] "Peptide.count"
##  [3] "Unique.peptides"
##  [4] "Confidence.score"
##  [5] "Anova..p."
##  [6] "Max.fold.change"
##  [7] "Highest.mean.condition"
##  [8] "Lowest.mean.condition"
##  [9] "Description"
## [10] "X20161007_EWM47_ER_50ng_1_5.35_7uL"
## [11] "X20161007_EWM47_ER_50ng_2_5.35_7uL"
## [12] "X20161007_EWM47_ER_50ng_3_5.35_7uL"
## [13] "X20161007_EWM47_ER_100ng_1_5.35_7uL"
## [14] "X20161007_EWM47_ER_100ng_2_5.35_7uL"
## [15] "X20161007_EWM47_ER_100ng_3_5.35_7uL"
## [16] "X20161007_EWM47_ER_50ng_1_5.35_7uL.1"
## [17] "X20161007_EWM47_ER_50ng_2_5.35_7uL.1"
## [18] "X20161007_EWM47_ER_50ng_3_5.35_7uL.1"
## [19] "X20161007_EWM47_ER_100ng_1_5.35_7uL.1"
## [20] "X20161007_EWM47_ER_100ng_2_5.35_7uL.1"
## [21] "X20161007_EWM47_ER_100ng_3_5.35_7uL.1"
```

FASTA database

```
database <- readAAStringSet("./20180502_Cr_mt_chl_yeast.fasta")
```

```r
# Split header by first space
names(database) <- sapply(strsplit(names(database), " "), head, 1)
```

```
##   A AAStringSet instance of length 26324
##         width seq                                     names
##     [1]   155 MDATSKADLPDYAADNRLP...SAATPYTTTQSPPSTTLS Cre38.g759997.t1....
##     [2]   575 MGGAKRRRGATAEDPETPG...DRGKYSADGVTVCGRGLH Cre08.g363350.t1....
##     [3]   664 MEAPTQRICRVCARQYARY...EASGGGGPNWRPGDVRVQ Cre08.g379050.t1....
##     [4]  3975 MEPSITRLTASARDRVGDR...QLAPPGAAGGPQLTGTAV Cre08.g364751.t1....
##     [5]   318 MQVSRAAVCARSSFVRVQA...ARAKSVFQEIAASAPKQA Cre08.g372000.t1....
##     ...   ... ...
## [26320]  1076 MRSFIKAHKKSTSFDESPK...LSPIQETPSSVQSSPKRA sp|P40021|ZRG8_YEAST
## [26321]   605 METPQMNAIQEEDNLSPEV...CIQTSIPTCETTIDIDRI sp|P53735|ZRG17_Y...
## [26322]   422 MVDLIARDDSVDTCQASNG...YLIMCCGAALMALLGKWA sp|Q12436|ZRT2_YEAST
## [26323]   433 MFSLPTLTSDITVEVNSSA...KTIVDSGKLPSSLLSYFV sp|P32527|ZUO1_YEAST
## [26324]   376 MSNVTTPWWKQWDPSEVTL...VICTLFGAGIMALIGKWA sp|P32804|ZRT1_YEAST
```

Define conditions/samples

```r
A <- 19:21
B <- 22:24

group <- list(A, B)
```

```
## [[1]]
## [1] 19 20 21
##
## [[2]]
## [1] 22 23 24
```

Filter score & contaminants

- Remove peptide identifications with Mascot score < 13 and any cRAP matches

```r
pepm <- pepm %>%
  filter_score() %>%
  filter_contaminants()
```

```
## Observations: 3,752
## Variables: 36
## $ X.                               <int> 125, 219, 286, 338, 4719...
## $ Retention.time..min.             <dbl> 26.06338, 27.22773, 31.4...
## $ Charge                           <int> 2, 2, 2, 2, 3, 3, 2, 2, ...
## $ m.z                              <dbl> 360.2119, 570.2643, 664....
## $ Measured.mass                    <dbl> 718.4093, 1138.5141, 132...
## $ Mass.error..u.                   <dbl> 0.004589289, 0.006438876...
## $ Mass.error..ppm.                 <dbl> 6.388167, 5.655540, -1.1...
## $ Score                            <dbl> 35.97, 93.38, 110.26, 10...
## $ Sequence                         <chr> "LAACLTK", "FCSDVTPGSAR"...
## $ Modifications                    <chr> "", "", "", "", "", "", ...
## $ Accession                        <chr> "Cre01.g005050.t1.1|PACi...
## $ Description                      <chr> "None", "None", "None", ...
## $ Use.in.quantitation              <chr> "True", "False", "True",...
```

```
## $ Max.fold.change                       <dbl> 1.098258, 1.117718, 1.06...
## $ Highest.mean.condition                <chr> "Condition 1", "Conditio...
## $ Lowest.mean.condition                 <chr> "Condition 2", "Conditio...
## $ Anova                                 <dbl> 4.150771e-02, 3.566783e-...
## $ Maximum.CV                            <dbl> 4.673430, 5.170363, 4.25...
## $ X20161007_EWM47_ER_50ng_1_5.35_7uL    <dbl> 5626.066461, 6268.403819...
## $ X20161007_EWM47_ER_50ng_2_5.35_7uL    <dbl> 5612.4415, 6268.6146, 77...
## $ X20161007_EWM47_ER_50ng_3_5.35_7uL    <dbl> 5176.50043, 5723.41512, ...
## $ X20161007_EWM47_ER_100ng_1_5.35_7uL   <dbl> 5130.12890, 5648.65218, ...
## $ X20161007_EWM47_ER_100ng_2_5.35_7uL   <dbl> 4866.0094, 5330.2781, 74...
## $ X20161007_EWM47_ER_100ng_3_5.35_7uL   <dbl> 4950.26209, 5358.31434, ...
## $ X20161007_EWM47_ER_50ng_1_5.35_7uL.1  <dbl> 4997.6920035, 5568.28680...
## $ X20161007_EWM47_ER_50ng_2_5.35_7uL.1  <dbl> 4897.4289, 5470.0070, 67...
## $ X20161007_EWM47_ER_50ng_3_5.35_7uL.1  <dbl> 4539.97630, 5019.64007, ...
## $ X20161007_EWM47_ER_100ng_1_5.35_7uL.1 <dbl> 5130.12890, 5648.65218, ...
## $ X20161007_EWM47_ER_100ng_2_5.35_7uL.1 <dbl> 4762.20374, 5216.56825, ...
## $ X20161007_EWM47_ER_100ng_3_5.35_7uL.1 <dbl> 4530.36973, 4903.81007, ...
## $ X20161007_EWM47_ER_50ng_1_5.35_7uL.2  <int> 4, 4, 2, 8, 0, 4, 1, 6, ...
## $ X20161007_EWM47_ER_50ng_2_5.35_7uL.2  <int> 5, 5, 2, 8, 0, 2, 2, 5, ...
## $ X20161007_EWM47_ER_50ng_3_5.35_7uL.2  <int> 5, 5, 2, 7, 0, 3, 1, 5, ...
## $ X20161007_EWM47_ER_100ng_1_5.35_7uL.2 <int> 4, 5, 3, 9, 1, 1, 2, 5, ...
## $ X20161007_EWM47_ER_100ng_2_5.35_7uL.2 <int> 5, 4, 2, 8, 0, 2, 1, 5, ...
## $ X20161007_EWM47_ER_100ng_3_5.35_7uL.2 <int> 4, 4, 2, 7, 0, 1, 2, 6, ...
```

Summarize duplicate features

- There were instances of rows with duplicated peak features and differing peptide identifications:
  - Some features were matched with peptides having identical sequence, modifications, and score, but alternate protein accessions. These groups were reduced to satisfy the principle of parsimony and represented by the protein accession with the highest number of unique peptides, else the protein with the largest confidence score assigned by Progenesis.
  - Some features were also duplicated with differing peptide identifications and were reduced to a single peptide with the highest Mascot ion score.

```r
pepm <- pepm %>%
  protm_map(protm) %>%
  pepm_reduce()
```

```
## Observations: 3,547
## Variables: 3
## $ Peptide.count    <int> 3, 5, 12, 4, 8, 8, 8, 6, 12, 5, 1, 12, 2, 10,...
## $ Unique.peptides  <int> 3, 5, 12, 4, 8, 8, 8, 5, 12, 5, 1, 12, 2, 10,...
## $ Confidence.score <dbl> 343.90, 432.61, 1348.19, 311.28, 418.10, 447....
```

Filter for modified peptides

- Results were limited to only peptides with one or more Cys-sites of reversible oxidation, defined here as the absence of carbamidomethylation on at least one cysteine residue in the peptide sequence.
- An identifier was then made by joining the protein accession of each feature with the particular site of Cys-oxidation in the protein sequence.

```r
pepm <- pepm %>%
  filter_oxidation(reduced = "IAM") %>%
  get_identifier_redox(database, reduced = "IAM")
```

```
## Observations: 3,162
## Variables: 40
## $ X.                                 <int> 2, 4, 6, 9, 10, 12, 14, ...
## $ Retention.time..min.               <dbl> 33.04547, 22.68870, 26.7...
## $ Charge                             <int> 2, 2, 2, 2, 2, 2, 2, 2, ...
## $ m.z                                <dbl> 654.3636, 582.2926, 438....
## $ Measured.mass                      <dbl> 1306.7127, 1162.5706, 87...
## $ Mass.error..u.                     <dbl> 0.006035954, 0.005432857...
## $ Mass.error..ppm.                   <dbl> 4.6192109, 4.6731637, 4....
## $ Score                              <dbl> 99.84, 98.09, 50.88, 89....
## $ Sequence                           <chr> "AAVVPGQAVAPPCK", "QVLCS...
## $ Modifications                      <chr> "", "", "", "", "", "", ...
## $ Accession                          <chr> "Cre06.g278213.t1.1|PACi...
## $ Description                        <chr> "light-harvesting chloro...
## $ Use.in.quantitation                <chr> "True", "True", "True", ...
## $ Max.fold.change                    <dbl> 1.061832, 1.075141, 1.10...
## $ Highest.mean.condition             <chr> "Condition 1", "Conditio...
## $ Lowest.mean.condition              <chr> "Condition 2", "Conditio...
## $ Anova                              <dbl> 1.491881e-01, 1.696374e-...
## $ Maximum.CV                         <dbl> 5.640156, 6.954685, 6.79...
## $ X20161007_EWM47_ER_50ng_1_5.35_7uL    <dbl> 333360.35597, 101765.491...
## $ X20161007_EWM47_ER_50ng_2_5.35_7uL    <dbl> 314066.08, 105604.53, 46...
## $ X20161007_EWM47_ER_50ng_3_5.35_7uL    <dbl> 297861.33672, 92128.0148...
## $ X20161007_EWM47_ER_100ng_1_5.35_7uL   <dbl> 300330.94, 93130.29, 420...
## $ X20161007_EWM47_ER_100ng_2_5.35_7uL   <dbl> 295195.48, 90858.40, 381...
## $ X20161007_EWM47_ER_100ng_3_5.35_7uL   <dbl> 294715.85, 94577.67, 386...
## $ X20161007_EWM47_ER_50ng_1_5.35_7uL.1  <dbl> 296127.39147, 90399.3197...
## $ X20161007_EWM47_ER_50ng_2_5.35_7uL.1  <dbl> 274054.76, 92150.75, 405...
## $ X20161007_EWM47_ER_50ng_3_5.35_7uL.1  <dbl> 261235.06177, 80799.5690...
## $ X20161007_EWM47_ER_100ng_1_5.35_7uL.1 <dbl> 300330.94, 93130.29, 420...
## $ X20161007_EWM47_ER_100ng_2_5.35_7uL.1 <dbl> 288898.13, 88920.13, 373...
## $ X20161007_EWM47_ER_100ng_3_5.35_7uL.1 <dbl> 269717.38, 86555.38, 353...
## $ X20161007_EWM47_ER_50ng_1_5.35_7uL.2  <int> 16, 10, 9, 10, 8, 10, 6,...
## $ X20161007_EWM47_ER_50ng_2_5.35_7uL.2  <int> 15, 10, 9, 9, 8, 11, 5, ...
## $ X20161007_EWM47_ER_50ng_3_5.35_7uL.2  <int> 18, 10, 8, 9, 6, 9, 6, 1...
## $ X20161007_EWM47_ER_100ng_1_5.35_7uL.2 <int> 16, 10, 8, 9, 8, 12, 6, ...
## $ X20161007_EWM47_ER_100ng_2_5.35_7uL.2 <int> 17, 10, 8, 8, 6, 12, 5, ...
## $ X20161007_EWM47_ER_100ng_3_5.35_7uL.2 <int> 16, 9, 7, 9, 7, 12, 5, 1...
## $ Peptide.count                      <int> 3, 5, 12, 4, 8, 8, 8, 6,...
## $ Unique.peptides                    <int> 3, 5, 12, 4, 8, 8, 8, 5,...
## $ Confidence.score                   <dbl> 343.90, 432.61, 1348.19,...
## $ Identifier                         <chr> "Cre06.g278213.t1.1|PACi...
```

```
## Observations: 3,162
## Variables: 1
## $ Identifier <chr> "Cre06.g278213.t1.1|PACid:30779171|--233", "Cre09.g...
```

Summarize duplicate identifiers

- The dataset was then reduced to unique identifiers by summing the abundance of all contributing features (*i.e.*, peptide charge states, missed cleavages, and combinations of additional variable modifications).
- Each identifier group was represented by the peptide with the highest Mascot score in the final dataset.

```
pepm <- pepm %>%
  identifier_reduce(group)
```

```
## Observations: 2,235
## Variables: 40
## $ X.                                <int> 9036, 6619, 5009, 7553, ...
## $ Retention.time..min.              <dbl> 29.83542, 17.89253, 29.5...
## $ Charge                            <int> 2, 2, 2, 2, 2, 2, 2, 2, ...
## $ m.z                               <dbl> 613.7781, 404.1883, 529....
## $ Measured.mass                     <dbl> 1225.5417, 806.3621, 105...
## $ Mass.error..u.                    <dbl> 0.011934976, 0.002898758...
## $ Mass.error..ppm.                  <dbl> 9.738626, 3.594871, 1.36...
## $ Score                             <dbl> 18.79, 26.49, 40.97, 44....
## $ Sequence                          <chr> "NWYQTQQCR", "EIEGCTR", ...
## $ Modifications                     <chr> "", "", "", "", "", "", ...
## $ Accession                         <chr> "Cre01.g000450.t1.2|PACi...
## $ Description                       <chr> "xyloglucanase 113", "No...
## $ Use.in.quantitation               <chr> "True", "True", "True", ...
## $ Max.fold.change                   <dbl> 13.391825, 1.093414, 23....
## $ Highest.mean.condition            <chr> "Condition 1", "Conditio...
## $ Lowest.mean.condition             <chr> "Condition 2", "Conditio...
## $ Anova                             <dbl> 2.920239e-02, 3.127701e-...
## $ Maximum.CV                        <dbl> 98.158412, 2.119884, 96....
## $ X20161007_EWM47_ER_50ng_1_5.35_7uL    <dbl> 77.356972, 246.630603, 6...
## $ X20161007_EWM47_ER_50ng_2_5.35_7uL    <dbl> 78.11523, 254.84021, 43....
## $ X20161007_EWM47_ER_50ng_3_5.35_7uL    <dbl> 76.35217, 244.99773, 69....
## $ X20161007_EWM47_ER_100ng_1_5.35_7uL   <dbl> 11.32172, 225.37112, 132...
## $ X20161007_EWM47_ER_100ng_2_5.35_7uL   <dbl> 5.989171, 226.677482, 11...
## $ X20161007_EWM47_ER_100ng_3_5.35_7uL   <dbl> 0.00000, 230.64678, 117....
## $ X20161007_EWM47_ER_50ng_1_5.35_7uL.1  <dbl> 68.7169841, 219.0844709,...
## $ X20161007_EWM47_ER_50ng_2_5.35_7uL.1  <dbl> 68.163525, 222.374134, 0...
## $ X20161007_EWM47_ER_50ng_3_5.35_7uL.1  <dbl> 66.963591, 214.871788, 8...
## $ X20161007_EWM47_ER_100ng_1_5.35_7uL.1 <dbl> 11.321716, 225.371118, 1...
## $ X20161007_EWM47_ER_100ng_2_5.35_7uL.1 <dbl> 5.861405, 221.841813, 11...
## $ X20161007_EWM47_ER_100ng_3_5.35_7uL.1 <dbl> 0.000000, 211.082804, 10...
## $ X20161007_EWM47_ER_50ng_1_5.35_7uL.2  <int> 1, 2, 0, 1, 1, 2, 0, 0, ...
## $ X20161007_EWM47_ER_50ng_2_5.35_7uL.2  <int> 1, 1, 0, 1, 1, 2, 0, 0, ...
## $ X20161007_EWM47_ER_50ng_3_5.35_7uL.2  <int> 1, 2, 0, 1, 2, 2, 1, 0, ...
## $ X20161007_EWM47_ER_100ng_1_5.35_7uL.2 <int> 0, 2, 1, 2, 2, 3, 1, 1, ...
## $ X20161007_EWM47_ER_100ng_2_5.35_7uL.2 <int> 0, 1, 0, 2, 2, 3, 1, 1, ...
## $ X20161007_EWM47_ER_100ng_3_5.35_7uL.2 <int> 0, 2, 0, 2, 3, 3, 1, 1, ...
## $ Peptide.count                     <int> 1, 1, 1, 5, 5, 5, 2, 6, ...
## $ Unique.peptides                   <int> 1, 1, 1, 5, 5, 5, 1, 6, ...
## $ Confidence.score                  <dbl> 18.79, 26.49, 61.71, 248...
## $ Identifier                        <chr> "Cre01.g000450.t1.2|PACi...
```

Select necessary variables

- Reduce variables to simplify in downstream processing
- Replace variable names with simple 'Letter-Number' format

```
pepm <- pepm %>%
  lfq_simplify(group)

colnames(pepm) <- get_design(group) %>%
  c("Identifier", .)
```

```
## Observations: 2,235
## Variables: 7
## $ Identifier <chr> "Cre01.g000450.t1.2|PACid:30788617|--321", "Cre01.g...
## $ `A-1`      <dbl> 77.356972, 246.630603, 60.088514, 90.801864, 932.04...
## $ `A-2`      <dbl> 78.11523, 254.84021, 43.22155, 74.85427, 1095.65775...
## $ `A-3`      <dbl> 76.35217, 244.99773, 69.93231, 55.07086, 925.35197,...
## $ `B-1`      <dbl> 11.32172, 225.37112, 132.83316, 96.82057, 1179.0715...
## $ `B-2`      <dbl> 5.989171, 226.677482, 113.182710, 90.135085, 1376.8...
## $ `B-3`      <dbl> 0.00000, 230.64678, 117.90911, 76.67195, 1334.62526...
```
