# Supplementary figures and images for: Evaluation of linear models and missing value imputation for the analysis of peptide-centric proteomics
Source: BMC Bioinformatics. 2019 Mar 14;20(Suppl 2):102. doi: 10.1186/s12859-019-2619-6 (PMC6419331; doi:10.1186/s12859-019-2619-6)

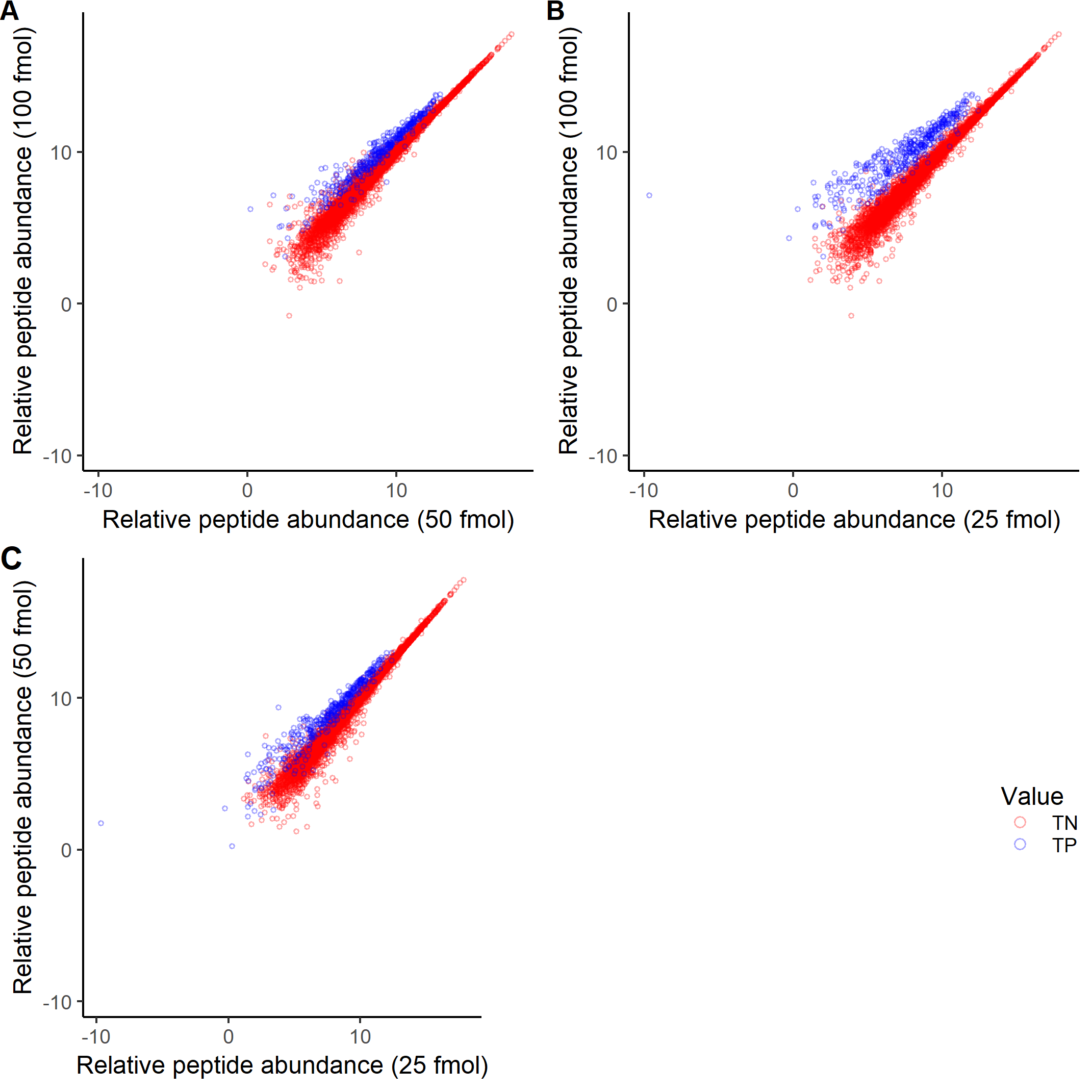

Supplement: Supplementary file 3 — Figure S1. Scatterplots of the UPS1 dataset showing the position of the true positives and the true negatives. A shows a comparison between 100 fmol (y-axis) and 25 fmol (x-axis; fold change of 4) spiked-in UPS1 protein. B shows a comparison between 100 fmol (y-axis) and 50 fmol (x-axis) and C shows comparisons of between 50 fmol (y-axis) and 25 fmol (x-axis; both comparisons having a fold change of 2). Each circle represents the mean of all replicates after running our imputation one time. True negatives (TN) was marked in red and true positives (TP) was marked in blue. All TN were Chlamydomonas peptides. (TIFF 166 kb) [file 12859_2019_2619_MOESM3_ESM.tiff]

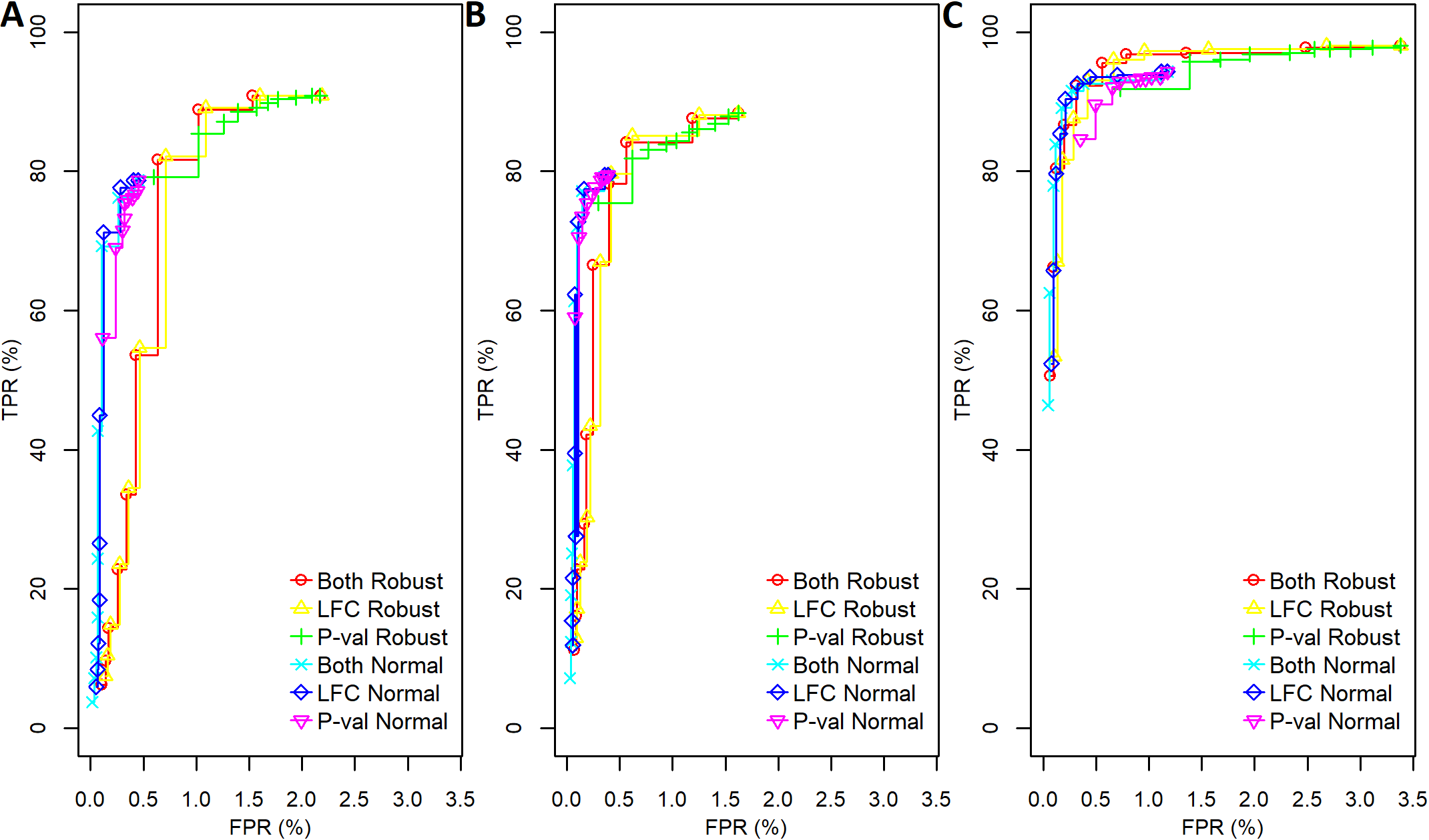

Supplement: Supplementary file 4 — Figure S2. ROC curves comparison of using limma with or without robust regression in our pipeline with the UPS1 dataset. The A-B show ROC curves of comparisons with a fold change of 2 (LFC of 1), A shows comparison between 100 fmol and 50 fmol, and B shows comparison between 50 fmol and 25 fmol. C shows a comparison with a fold change of 4 (LFC of 2) comparing the lowest and highest spike-in concentrations. Both, LFC, and P-val indicate which parameters were varied (p-value and LFC at the same time, LFC only, and p-value only, respectively) when creating the ROC curves. LFC was changed from zero to two and p-value from 0.05 to 0.001, simultaneously or separately. When only LFC was changed the p-value was fixed to 0.05, and when the p-value was changed LFC was fixed to zero. Lower LFC cut-off was set to –infinity as there was no TP with a decreasing fold change. “Robust” indicates limma with robust regression and “Normal” indicates limma using normal regression. Y-axis show TPR and x-axis show FPR expressed as a percentage (TPR*100, FPR*100, respectively). (TIFF 362 kb) [file 12859_2019_2619_MOESM4_ESM.tiff]

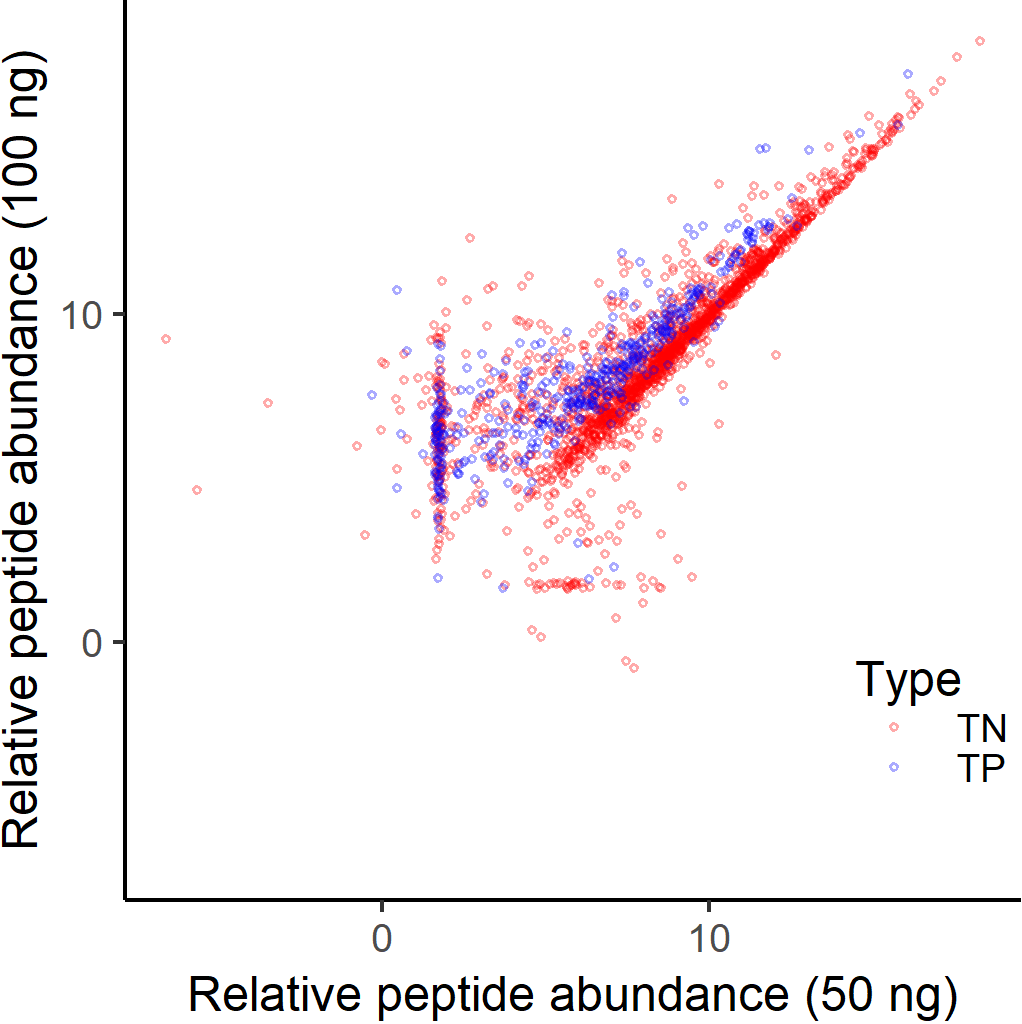

Supplement: Supplementary file 5 — Figure S3. Scatterplot of the yeast dataset showing the position of the true positives and the true negatives. Comparison between 100 ng (y-axis) and 50 ng (x-axis) yeast protein spike-in enriched for reversibly oxidized cysteines, in a background of Chlamydomonas reinhardtii lysate. Each dot represents the mean of all replicates after running our imputation one time. True negatives (TN) was marked in red and true positives (TP) was marked in blue. (TIFF 172 kb) [file 12859_2019_2619_MOESM5_ESM.tiff]

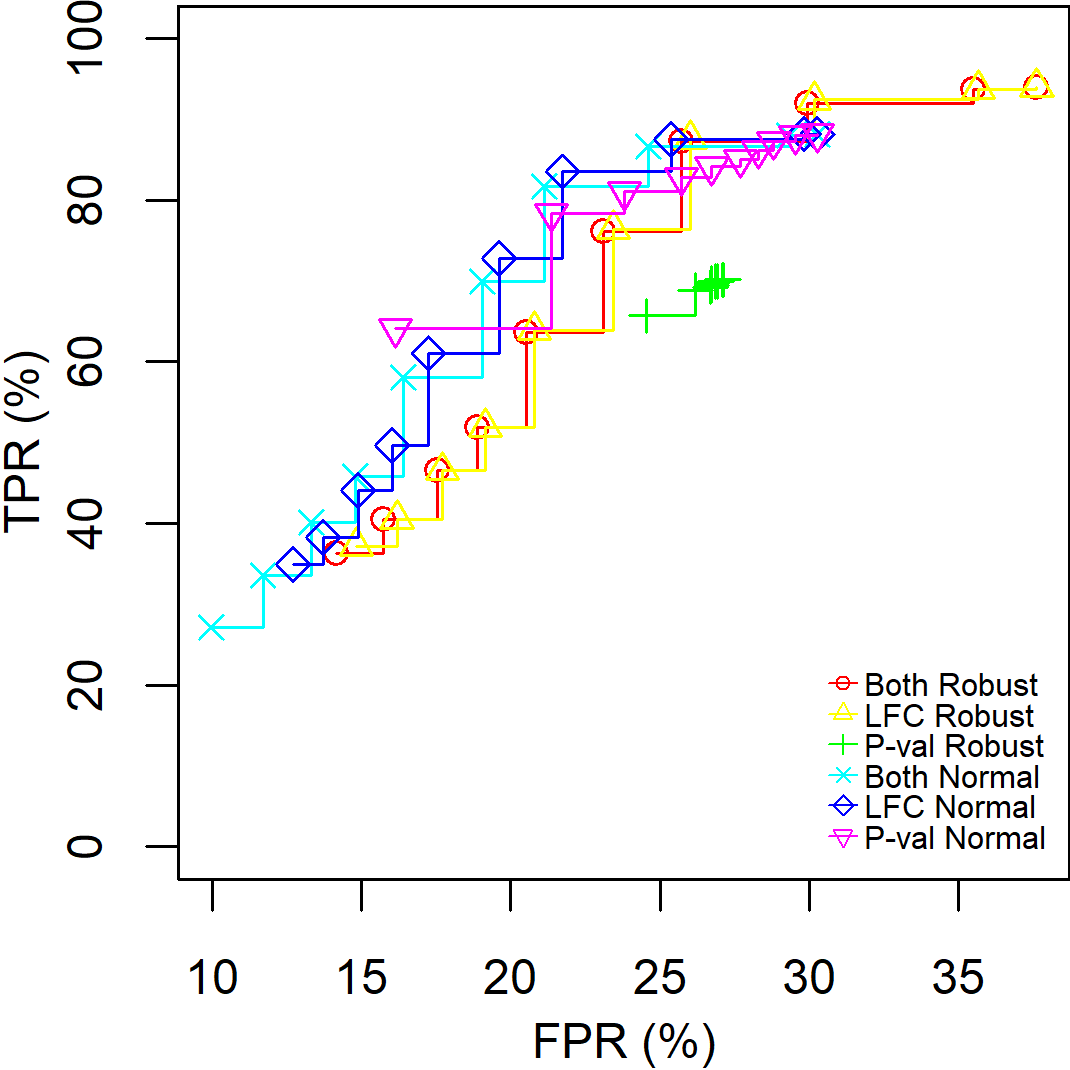

Supplement: Supplementary file 6 — Figure S4. ROC curves comparison of using limma with or without robust regression in our pipeline with the yeast dataset. Both, LFC, and P-val indicates which parameters were varied (p-value and LFC at the same time, LFC only, and p-value only, respectively) when creating the ROC curves. LFC was changed from zero to two and p-value from 0.05 to 0.001, simultaneously or separately. When only LFC was changed the p-value was fixed to 0.05, and when the p-value was changed LFC was fixed to zero. Lower LFC cut-off was set to –infinity as there was no TP with a decreasing fold change. “Robust” indicates limma with robust regression and “Normal” indicates limma using normal regression. Y-axis show TPR and x-axis show FPR expressed as a percentage (TPR*100, FPR*100, respectively). (TIFF 39 kb) [file 12859_2019_2619_MOESM6_ESM.tiff]
