# Supplementary material for: Evaluation of linear models and missing value imputation for the analysis of peptide-centric proteomics
Source: BMC Bioinformatics. 2019 Mar 14;20(Suppl 2):102. doi: 10.1186/s12859-019-2619-6 (PMC6419331; doi:10.1186/s12859-019-2619-6)
Supplement: Supplementary file 8 — File containing a tutorial on using the computational pipeline. (PDF 1543 kb) [file 12859_2019_2619_MOESM8_ESM.pdf]

Tutorial to Evaluation of linear models and missing value imputation for the analysis of peptide- centric proteomics

Philip Berg

May 22, 2018

Contents

Loading scripts **1**

Loading data and its required structure **2**

Running the pipeline **2**

Creating ROC curves **5**

The pipeline . . . 5
t-test . . . 6
Plotting the curves . . . 7
Random forest . . . 8

Loading scripts

The pipeline currently offers the possibility to receive an email when a run is finished. For this to function, the `Mailer.R` scripts needs to be properly configured. Opening the script will give instruction for how this is done. No error will occur from skipping this step. The easiest way to load the scripts is to use the `R.utils` package. First we will load another package, `pacman` which is used in the pipeline for managing packages. It can be loaded and/or installed with the following code:

```
if(!("pacman" %in% .packages(all.available = T))){
    install.packages("pacman")
    library("pacman")
  }else if(!("pacman" %in% (.packages()))){
    library("pacman")
  }
```

we can then install and/or load `R.utils` with `p_load`:

```
p_load(R.utils)
```

From `R.utils` we can read in the pipeline with the `sourceDirectory` function.

```
sourceDirectory("Scripts/")
```

Do note that the user has to specify the correct directory to the repository of functions used in the pipeline. Here, we assume that the *Scripts* folder is in the working directory.

Loading data and its required structure

Here we start by using the UPS1 dataset as an example. We start by reading in the data using `read.csv` and store it in a variable.

```
Data <- read.csv(file = "UPS1.csv", stringsAsFactors = F)
```

We can look at the needed structure of the data with `str`.

```
str(Data)
```

```
## 'data.frame':    10962 obs. of  13 variables:
##  $ Identifier: chr  "1::Cre01.g000350.t1.1|PACid:30788481|--AVLLFATGSGISPLR" "1::Cre01.g000350.t1.1|I
##  $ X25.1     : num  695.2 736 680.9 491.3 7.9 ...
##  $ X25.2     : num  673.7 636.24 726.96 447.56 1.19 ...
##  $ X25.3     : num  689 657 638 463 0 ...
##  $ X25.4     : num  694.1 607.6 646.6 443.7 26.9 ...
##  $ X50.1     : num  741 793 732 372 0 ...
##  $ X50.2     : num  738.8 782.7 725.6 408.3 33.4 ...
##  $ X50.3     : num  733 694 654 408 0 ...
##  $ X50.4     : num  796 734 661 475 0 ...
##  $ X100.1    : num  644 654 628 413 0 ...
##  $ X100.2    : num  675 642 694 389 0 ...
##  $ X100.3    : num  715 619 607 476 0 ...
##  $ X100.4    : num  735 613 684 433 0 ...
```

There has to be a column called Identifier which holds an unique ID for each peptide, and then each condition has to have the same name and end with a unique number for each replicate.

Running the pipeline

First, *variance-stabilizing* transformation is done by converting the raw data to $\log_2$ scale. This can be done with the `Log_Transformer` function in the pipeline.

```
log_data <- Log_Transformer(Data)
```

After this, there is a need to define which comparisons to be made. There is three different conditions, **X25**, **X50**, and **X100**. The comparisons are defined by making a matrix with two rows, where each number in each column is compared. Each number corresponds to the j:th condition in the order they occur in the dataframe. If we want to do all possible comparisons we would define the following matrix:

```
comp <- matrix(c(2,1,#Comparing X50 with X25
                 3,2,#Comparing X100 with X50
                 3,1),#Comparing X100 with X25
                nrow = 2)
#The output is as follows:
comp
```

```
##      [,1] [,2] [,3]
## [1,]    2    3    3
## [2,]    1    2    1
```

We are then ready to run the pipeline. This can be done with the `multiple_imputation` function.

```r
Sig_hits <- multiple_imputation(Data = log_data,#adding the transformed data
                                #as the dataset to run the analysis on
                                Pre_cleaned = F,#We did not remove outliers before running
                                                #this function (F is the standard setting)
                                n = 100,#Running 20 multiple imputations
                                Comparisons = comp,#Adding which comparisons to run.
                                                   #As defined above in this case.
                                FDR_Criteria = 0.05,#Using an alpha level of 0.05
                                                    #(0.05 is the standard setting)
                                LFC_limits = c(-Inf,1),#all true positives has an increase
                                                       #in log fold change, hence we set
                                                       #the lower cut-off to -Inf
                                Binom_Prob = 1/2,#The propability hypothesis for the
                                                 #binomial test
                                Robust = F,#Runnig limma with normal (least squares)
                                           #regression
                                verbose = T)#Print progress of the pipeline
```

```
## Generating 100 imputed datasets.

## Multiple data imputation: 79.02 sec elapsed

## Running LIMMA

## LIMMA: 13.67 sec elapsed

## Extracting FDR corrected p-value from LIMMA and calculating LFC.

## Limma FDR extraction and LFC calculation: 108.39 sec elapsed
## Significance: 122.06 sec elapsed

## Calculating 5:th and 95:th percentile, median, and mean for FDR and LFC.

## Descriptive statistics: 28.14 sec elapsed

## Running binomial test.

## Calculate binomial p-value: 7.06 sec elapsed

## Plotting results

## Saving 13 x 9 in image
## Saving 13 x 9 in image
## Saving 13 x 9 in image

## Plot results: 4.44 sec elapsed

## Saving results

## Save results: 0.03 sec elapsed
## Total: 241.37 sec elapsed

## Sending email

## NULL

## No internet or Mailer not setup; skipping email
```

```r
#The output from multiple_imputation() contains a summary dataset with the 95:th and
#5:th percentile, median, and mean of the p-value from limma and the LFC for the different
#imputations.
#It also contains the number of missing values each feature contained in each condition
```

```r
#The categorize can be seen with head of the dataset:
str(Sig_hits[['Data_Summary']])
```

```
## 'data.frame':    10959 obs. of  40 variables:
##  $ Identifier               : chr  "1::Cre01.g000350.t1.1|PACid:30788481|--AVLLFATGSGISPLR" "1::Cre
##  $ X25.1                    : num  9.44 9.52 9.41 8.94 2.98 ...
##  $ X25.2                    : num  9.396 9.313 9.506 8.806 0.255 ...
##  $ X25.3                    : num  9.43 9.36 9.32 8.86 2.57 ...
##  $ X25.4                    : num  9.44 9.25 9.34 8.79 4.75 ...
##  $ X50.1                    : num  9.53 9.63 9.52 8.54 4.93 ...
##  $ X50.2                    : num  9.53 9.61 9.5 8.67 5.06 ...
##  $ X50.3                    : num  9.52 9.44 9.35 8.67 5.04 ...
##  $ X50.4                    : num  9.64 9.52 9.37 8.89 5.18 ...
##  $ X100.1                   : num  9.33 9.35 9.3 8.69 3.03 ...
##  $ X100.2                   : num  9.4 9.33 9.44 8.6 2.96 ...
##  $ X100.3                   : num  9.48 9.27 9.25 8.9 3 ...
##  $ X100.4                   : num  9.52 9.26 9.42 8.76 3.01 ...
##  $ X25._Missing             : num  0 0 0 0 1 0 0 0 0 0 ...
##  $ X50._Missing             : num  0 0 0 0 3 0 0 0 0 0 ...
##  $ X100._Missing            : num  0 0 0 0 4 0 0 0 0 0 ...
##  $ X50._Vs_X25._P_val_5Perc : num  0.336 0.247 0.827 0.533 0.115 ...
##  $ X50._Vs_X25._P_val_median : num  0.337 0.247 0.827 0.534 0.136 ...
##  $ X50._Vs_X25._P_val_95Perc : num  0.338 0.248 0.828 0.534 0.169 ...
##  $ X50._Vs_X25._P_val_mean   : num  0.337 0.248 0.827 0.534 0.14 ...
##  $ X50._Vs_X25._LFC_5Perc    : num  0.1279 0.19 0.0425 -0.1541 2.2966 ...
##  $ X50._Vs_X25._LFC_median   : num  0.1279 0.19 0.0425 -0.1541 2.413 ...
##  $ X50._Vs_X25._LFC_95Perc   : num  0.1279 0.19 0.0425 -0.1541 2.5005 ...
##  $ X50._Vs_X25._LFC_mean     : num  0.1279 0.19 0.0425 -0.1541 2.4036 ...
##  $ X100._Vs_X50._P_val_5Perc : num  0.4183 0.0809 0.8576 0.9861 0.1915 ...
##  $ X100._Vs_X50._P_val_median: num  0.4201 0.0814 0.8591 0.9867 0.242 ...
##  $ X100._Vs_X50._P_val_95Perc: num  0.4221 0.0818 0.86 0.9875 0.2937 ...
##  $ X100._Vs_X50._P_val_mean  : num  0.4201 0.0814 0.859 0.9868 0.2409 ...
##  $ X100._Vs_X50._LFC_5Perc   : num  -0.1207 -0.2477 -0.0857 0.0421 -2.2077 ...
##  $ X100._Vs_X50._LFC_median  : num  -0.1207 -0.2477 -0.0857 0.0421 -2.0848 ...
##  $ X100._Vs_X50._LFC_95Perc  : num  -0.1207 -0.2477 -0.0857 0.0421 -1.9652 ...
##  $ X100._Vs_X50._LFC_mean    : num  -0.1207 -0.2477 -0.0857 0.0421 -2.0832 ...
##  $ X100._Vs_X25._P_val_5Perc : num  0.965 0.722 0.795 0.552 0.819 ...
##  $ X100._Vs_X25._P_val_median: num  0.966 0.723 0.796 0.553 0.871 ...
##  $ X100._Vs_X25._P_val_95Perc: num  0.966 0.723 0.796 0.553 0.908 ...
##  $ X100._Vs_X25._P_val_mean  : num  0.966 0.723 0.796 0.553 0.869 ...
##  $ X100._Vs_X25._LFC_5Perc   : num  0.00718 -0.05764 -0.04324 -0.11197 0.23077 ...
##  $ X100._Vs_X25._LFC_median  : num  0.00718 -0.05764 -0.04324 -0.11197 0.31485 ...
##  $ X100._Vs_X25._LFC_95Perc  : num  0.00718 -0.05764 -0.04324 -0.11197 0.42931 ...
##  $ X100._Vs_X25._LFC_mean    : num  0.00718 -0.05764 -0.04324 -0.11197 0.32044 ...
```

The pipeline saves one figure and one tsv file for each comparison. The tsv files are saved in the working directory and the figures are made in the sub-directory */figures/Scatter significance*. They look as follows:

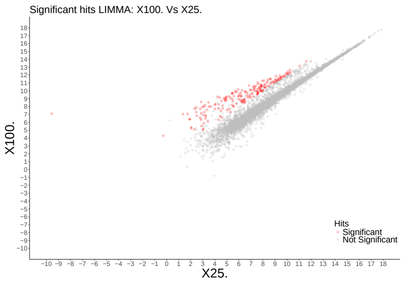

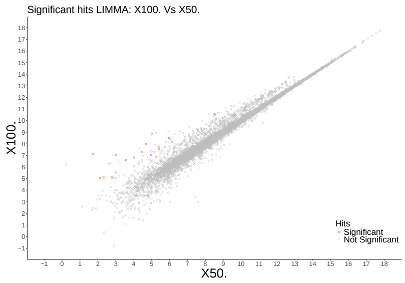

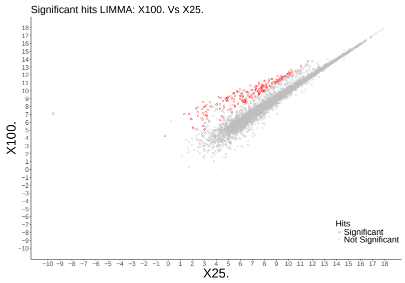

Creating ROC curves

The pipeline

To generate ROC curves there is a need to tweak the parameters. Here we exemplify by tweaking both the p-value and the $\log_2$ fold change (LFC) at the same time. We set up the parameters as follows:

```
#Sample 10 parameters between from 0.05 to 0.001
p_val <- seq(from = 0.05, to =  0.001, length.out = 10)
#Set lower limit to -Inf and then sample 10 paramters for upper limit between 0 and 2
LFC <- matrix(c(rep(-Inf, length.out = 10),
                seq(from = 0, to = 2, length.out = 10)), ncol = 2)
```

to get two evenly inter-spaced parameter sets. We can then loop over the different parameters and generate one result for each set.

```
Pipe_both <- NULL
for (i in seq_along(p_val)) {
  Pipe_both[[i]] <- multiple_imputation(Data = log_data,
                                        n = 100,
                                        Comparisons = comp,
                                        FDR_Criteria = p_val[i],
                                        LFC_limits = LFC[i,],
                                        verbose = F)#Silencing redundant messages of the
                                                    #progress of each run to not flood the
                                                    #tutorial.
}
```

To calculate the true positive rate (TPR) and the false positive rate (FPR) of each parameter set, we can use the `TPR_FDR` function in the pipeline.

```
ROC_pipe <- TPR_FPR(Data = log_data,#Note that we use the transformed data as it contains
                                    #all ID's. In case a TP were to be filtered out that
                                    #will penalize the pipeline.
                      Results = Pipe_both,#The results from tweaking the parameters
                    ID_TP = '_HUMAN')#Lastly a part of the ID for the TPs
#The output is then given as
lapply(ROC_pipe, head, 3)
```

```
## $FPR
##   X50._Vs_X25._FPR X100._Vs_X50._FPR X100._Vs_X25._FPR
## 1        0.3788238         0.4545885          1.174354
## 2        0.3598826         0.3882943          1.079648
## 3        0.1420589         0.2651766          0.653471
##
```

```
## $TPR
##   X50._Vs_X25._TPR X100._Vs_X50._TPR X100._Vs_X25._TPR
## 1         79.40447          78.66005          94.29280
## 2         79.15633          77.17122          93.54839
## 3         77.17122          76.17866          93.05211
```

t-test

For the t-test, we implemented a function called `My_ttest`, it runs on cleaned data. Data can be cleaned using the `Cleaner` function:

```
clean_data <- Cleaner(log_data, F)
```

```
## Replicate filter: 0.08 sec elapsed
## Missing annotation: 0.22 sec elapsed
## No id variables; using all as measure variables
## Imputation: 0.86 sec elapsed
## Data imputation: 0.93 sec elapsed
```

```
#The cleaner function also returns the number of missing values there was for a feature in
#a category
head(clean_data, 3)
```

```
##                                                        Identifier    X25.1
## 1         1::Cre01.g000350.t1.1|PACid:30788481|--AVLLFATGSGISPLR 9.441353
## 2           1::Cre01.g000350.t1.1|PACid:30788481|--GFALDRLPASTTR 9.523582
## 3 1::Cre01.g000350.t1.1|PACid:30788481|--VVIDVGAPLAAGYTVPGQFVQVK 9.411349
##      X25.2    X25.3    X25.4    X50.1    X50.2    X50.3    X50.4   X100.1
## 1 9.395968 9.428122 9.438970 9.532415 9.528966 9.517275 9.637467 9.331848
## 2 9.313426 9.359053 9.247015 9.631474 9.612256 9.439177 9.520306 9.352951
## 3 9.505738 9.317059 9.336818 9.516211 9.503058 9.352776 9.368944 9.295469
##     X100.2   X100.3   X100.4 X25._Missing X50._Missing X100._Missing
## 1 9.398251 9.480794 9.522248            0            0             0
## 2 9.325454 9.274017 9.260109            0            0             0
## 3 9.438726 9.246008 9.417799            0            0             0
```

. Then running the t-test with the same parameters as for the pipeline:

```
ttest_both <- NULL
for (i in seq_along(p_val)) {
  ttest_both[[i]] <- My_ttest(Data = clean_data,#Use the clean data
                              Pval = p_val[i],#Change the alpha levels as for the pipeline
                              LFC = LFC[i,2],#My_ttest only takes an upper LFC cut-off
                              Comparisons = comp)#The comparison to make, as before
}
```

. We get TPR and FPR just like for the pipeline

```
  ROC_ttest <- TPR_FPR(Data = log_data,
                      Results = ttest_both,
                      ID_TP = '_HUMAN')
lapply(ROC_ttest, head, 3)
```

```
## $FPR
##   X50.VsX25._FPR X100.VsX50._FPR X100.VsX25._FPR
## 1     0.23676485       0.4924709       1.1838242
```

```
## 2     0.13258831       0.2083531       0.4640591
## 3     0.08523534       0.1610001       0.3030590
##
## $TPR
##   X50.VsX25._TPR X100.VsX50._TPR X100.VsX25._TPR
## 1       47.39454        62.77916        82.13400
## 2       46.65012        62.28288        81.14144
## 3       45.65757        60.29777        79.40447
```

Plotting the curves

Each plot can then be made with the `ROC_Plotter` function.

```
  ROC_Plotter(ROC_pipe[[1]], ROC_ttest[[1]],
              ROC_pipe[[2]], ROC_ttest[[2]],#Add first TPR then FPR in the same order
              main = "Example figure")
legend("bottomright", inset = 0.01,
       legend = paste0(
         rep(c("Both"), times = 6),
         rep(c("_Lim_", "_ttest_"), each = 3),
         rep(c('50_vs_25', '100_vs_50', '100_vs_25'), times = 2)),
       col = rainbow(6), lty = 1, cex = 1,
       box.lty = 0, x.intersp = .25, y.intersp = 1,
       lwd = 1, pt.cex = 2, xpd = T, seg.len = 1, bg = "transparent",
       pch = 1:6)
```

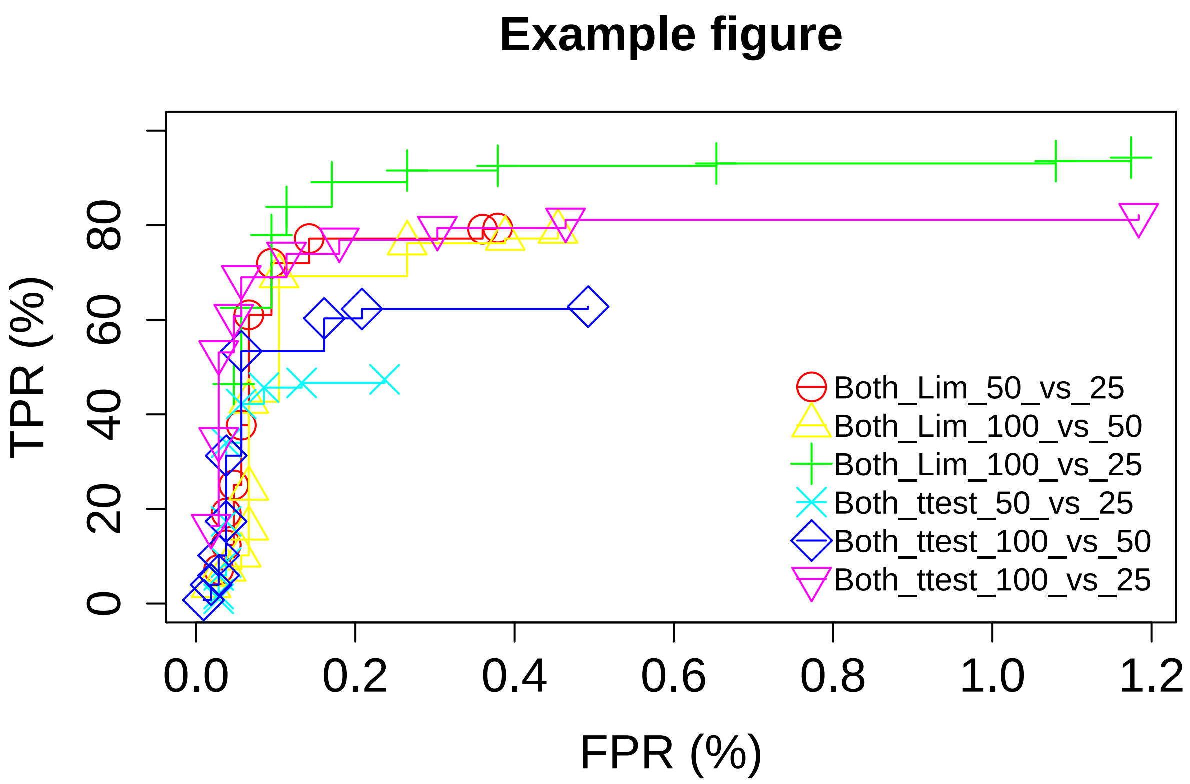

Random forest

For random forest imputation, we used the `rforimp` function. Here this will be examplified with the Yeast dataset. Which first has to be loaded and $\log_2$ transformed.

```
#Load data
Data <- read.csv(file = "Yeast.csv", stringsAsFactors = F)
#And log transform
log_data <- Log_Transformer(Data)
```

Then we can do imputation with `rforimp`

```
RF_imp <- rforimp(log_data)
```

```
##   missForest iteration 1 in progress...done!
##   missForest iteration 2 in progress...done!
##   missForest iteration 3 in progress...done!
##   missForest iteration 4 in progress...done!
##   missForest iteration 5 in progress...done!
##   missForest iteration 6 in progress...done!
##   missForest iteration 7 in progress...done!
##   missForest iteration 8 in progress...done!
##   missForest iteration 9 in progress...done!
##   missForest iteration 10 in progress...done!
##   missForest iteration 11 in progress...done!
```

```
#rforimp also returns the number of missing values in a condition and feature
```

We then have to run the data through limma using `My_limma`. And to get ROC curves we have to run it using different parameters.

```
#load needed packages
p_load('magrittr', 'tibble', 'dplyr', 'purrr')
#Setting up comparisons
comp <- matrix(c(2,1)#There is only one comparison in the Yeast dataset
               , nrow = 2)
#Generate datasets
RF_lim <- lapply(rep(list(RF_imp), length(p_val)),
                 MyLimma,
                 Comparisons =  comp,
                 Robust = F)
#Extract p-value from limma and calculate LFC
for (i in seq_along(RF_lim)) {
  RF_lim[[i]][[1]] <- RF_lim[[i]][[1]][5] %>%  #Only keep the adjusted p-val
    rownames_to_column(var = 'Identifier') %>% #MyLimma returns Identifiers as row names
                                               #Change rownames to Identifier column
    mutate(LFC = apply(RF_imp %>%#Add LFC
                  Data_Mean() %>% #Get the mean of each conditon
                 select(contains('mean')),
                 1, function(x) x[2]-x[1] #Calculate the LFC
                 ))
}
#Take the significant hits in each dataset
Subs <- function(x, p_val, LFC){
x[1]<p_val & x[2]>LFC
}
for (i in seq_along(RF_lim)) {
```

```
  Flag <- apply(RF_lim[[i]][[1]] %>% keep(is.numeric), 1,
                function(x) Subs(x, p_val = p_val[i], LFC = LFC[i,2]))
  RF_lim[[i]][[1]] <- subset(RF_lim[[i]][[1]],Flag)
}
```

And to compare with our method we run it like before:

```
Pipe_both <- NULL
for (i in seq_along(p_val)) {
  Pipe_both[[i]] <- multiple_imputation(Data = log_data,
                                        n = 100,
                                        Comparisons = comp,
                                        FDR_Criteria = p_val[i],
                                        LFC_limits = LFC[i,],
                                        verbose = F)
}
```

Finally, we get the TPR and FPR and make a plot:

```
ROC_RF <- TPR_FPR(Data = log_data,
                       Results = RF_lim,
                       ID_TP = '_YEAST')
ROC_pipe <- TPR_FPR(Data = log_data,
                    Results = Pipe_both,
                    ID_TP = '_YEAST')
  ROC_Plotter(ROC_pipe[[1]], ROC_RF[[1]],
              ROC_pipe[[2]], ROC_RF[[2]],
              main = "Example figure")
legend("bottomright", inset = 0.01,
       legend = paste0(
         rep(c("Both"), times = 2),
         rep(c("_Norm_", "_RF_"), each = 1),
         '50_vs_25'),
       col = rainbow(2), lty = 1, cex = 1,
       box.lty = 0, x.intersp = .25, y.intersp = 1,
       lwd = 1, pt.cex = 2, xpd = T, seg.len = 1, bg = "transparent",
       pch = 1:2)
```

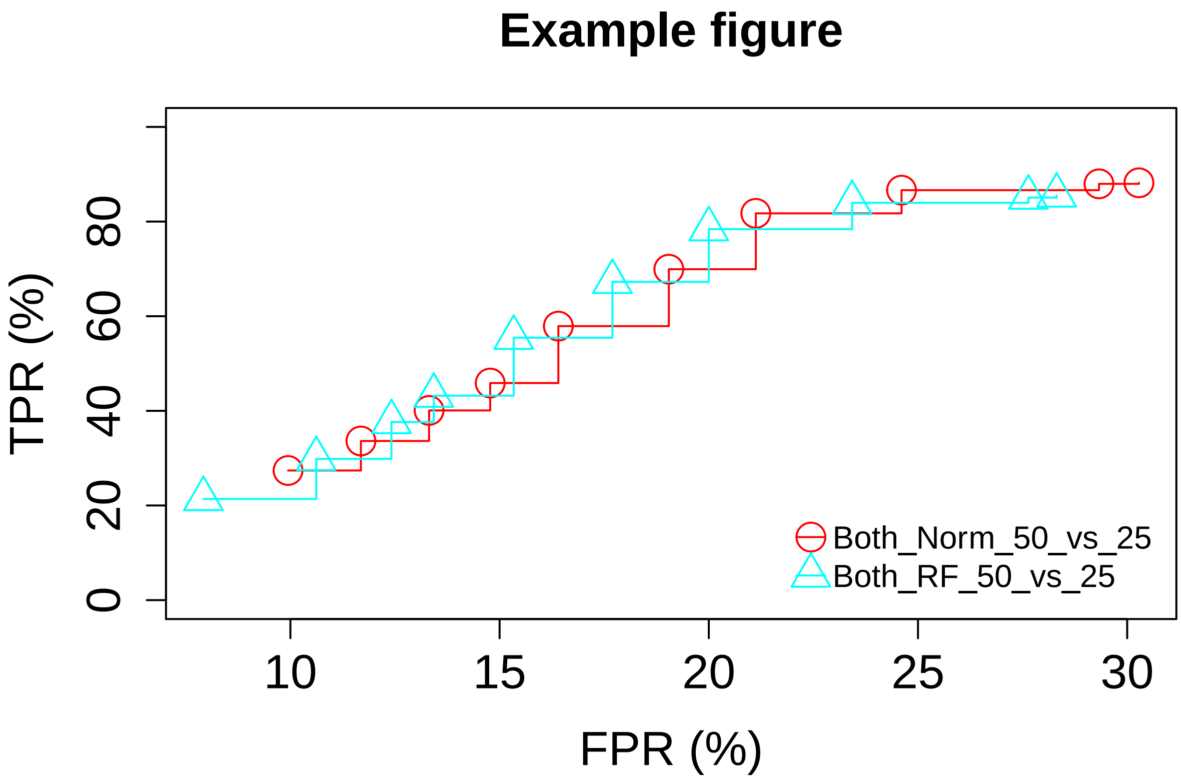
Example figure
TPR (%)
FPR (%)
0
20
40
60
80
10
15
20
25
30
Both_Norm_50_vs_25
Both_RF_50_vs_25
